# SAMHD1 Inhibits LINE-1 Retrotransposition by Promoting Stress Granule Formation

**DOI:** 10.1371/journal.pgen.1005367

**Published:** 2015-07-02

**Authors:** Siqi Hu, Jian Li, Fengwen Xu, Shan Mei, Yann Le Duff, Lijuan Yin, Xiaojing Pang, Shan Cen, Qi Jin, Chen Liang, Fei Guo

**Affiliations:** 1 MOH Key Laboratory of Systems Biology of Pathogens, Institute of Pathogen Biology, Chinese Academy of Medical Sciences & Peking Union Medical College, Beijing, P. R. China; 2 Lady Davis Institute, Jewish General Hospital, Montreal, Quebec, Canada; 3 Institute of Medicinal Biotechnology, Chinese Academy of Medical Sciences & Peking Union Medical College, Beijing, P. R. China; Fred Hutchinson Cancer Research Center, UNITED STATES

## Abstract

The SAM domain and HD domain containing protein 1 (SAMHD1) inhibits retroviruses, DNA viruses and long interspersed element 1 (LINE-1). Given that in dividing cells, SAMHD1 loses its antiviral function yet still potently restricts LINE-1, we propose that, instead of blocking viral DNA synthesis by virtue of its dNTP triphosphohydrolase activity, SAMHD1 may exploit a different mechanism to control LINE-1. Here, we report a new activity of SAMHD1 in promoting cellular stress granule assembly, which correlates with increased phosphorylation of eIF2α and diminished eIF4A/eIF4G interaction. This function of SAMHD1 enhances sequestration of LINE-1 RNP in stress granules and consequent blockade to LINE-1 retrotransposition. In support of this new mechanism of action, depletion of stress granule marker proteins G3BP1 or TIA1 abrogates stress granule formation and overcomes SAMHD1 inhibition of LINE-1. Together, these data reveal a new mechanism for SAMHD1 to control LINE-1 by activating cellular stress granule pathway.

## Author Summary

Long interspersed element 1 (LINE-1 or L1) comprises 17% of human genome, and has played a major role in shaping the evolution of human genome. Approximately 100 copies of LINE-1 are still active in an average individual genome. Movement of these LINE-1 sequences to new loci in the genome has the potential of causing sporadic cases of disease. Among the multi-layered mechanisms by which the host controls LINE-1 activity is a group of host restriction factors including APOBEC3 proteins. SAMHD1 was known for the association of its mutations with the Aicardi-Goutieres syndrome (AGS), a congenital autoimmune disease. SAMHD1 was recently reported as a host restriction factor that inhibits a number of retroviruses and DNA viruses including human immunodeficiency virus type 1 (HIV-1) and herpes simplex virus 1 (HSV-1). Here, we demonstrate that SAMHD1 inhibits LINE-1 retrotransposition through promoting the sequestration of LINE-1 RNP within the cytoplasmic stress granules. SAMHD1 promotes the formation of large stress granules by inducing phosphorylation of eIF2α and disrupting eIF4A/eIF4G interaction. This is the first report describing the role of SAMHD1 in modulating the formation of stress granules. We envision that this function of SAMHD1 not only contributes to the inhibition of LINE-1, but also the restriction of various viruses.

## Introduction

SAM domain and HD domain containing protein 1 (SAMHD1) was first identified as an interferonγ-induced protein in macrophages and dendritic cells, and was regarded as a negative regulator of cellular innate immunity [1]. Mutations in SAMHD1 were later found to associate with the Aicardi-Goutieres syndrome (AGS) [2], a congenital autoimmune disease that is characterized with low but persistent levels of type I interferon and inflammatory cytokines [3]. As a deoxynucleotide triphosphate (dNTP) triphosphohydrolase, SAMHD1 is able to decrease dNTP level in non-cycling cells below the threshold that is required for DNA synthesis [4,5]. By virtue of this function, SAMHD1 inhibits a number of retroviruses and DNA viruses including human immunodeficiency virus type 1 (HIV-1) and herpes simplex virus 1 (HSV-1) [6,7]. This mechanism of restriction is supported by the rescue of HIV-1 replication in SAMHD1-expressing cells with exogenous deoxynucleotides [8]. In turn, viruses have evolved countermeasures to evade SAMHD1 inhibition. HIV-2 and certain strains of simian immunodeficiency virus (SIV) carry an auxiliary protein called Vpx that is able to direct SAMHD1 to the E3 ligase complex Cul4/CRL4/DCAF1 and causes SAMHD1 ubiquitination and subsequent proteasomal degradation [9–17].

SAMHD1 is not as antiviral in cycling cells as in non-cycling cells. For example, both the activated and resting CD4^+^ T cells in the peripheral blood express similar levels of SAMHD1, yet activated T cells are fully susceptible to HIV-1 infection as opposed to the resistance of resting T cells [18,19]. In addition, a monocytic cell line called THP-1 becomes resistant to HIV-1 infection after differentiation into macrophages with PMA treatment [20], even though PMA treatment does not change the expression of SAMHD1 [20]. One mechanism behind this cell cycling-dependent antiviral phenotype of SAMHD1 involves phosphorylation of the T592 residue by the cyclin-dependent kinase 1 (CDK1) and cyclin A2 [20,21]. Cyclin A2 is a cell-cycle dependent kinase with the highest expression at S phase [22], whereas CDK1 is poorly expressed in differentiated cells [23–25]. It remains unclear how T592 phosphorylation regulates the antiviral activity of SAMHD1, since both the phosphorylated and the unphosphorylated SAMDH1 exhibit similar triphosphohydrolase activity *in vitro* [20,26].

Considering that the antiviral activity of SAMHD1 is impaired in cycling cells as a result of T592 phosphorylation, it is interesting to note that SAMHD1 potently diminishes the retrotransposition of an autonomous retroelement called LINE-1 (long interspersed element 1) in actively dividing HEK-293T and HeLa cells [27]. LINE-1 is a non-LTR (long terminal repeat) retrotransposon, comprises approximately 17% of human genome (reviewed in [28]). It carries two open reading frames (ORFs). ORF1p binds to LINE-1 RNA and forms RNA protein complex (RNP) [29–32]. ORF2p is an enzyme that has endonuclease and reverse transcriptase activities [33,34]. ORF2p is an essential component of LINE-1 RNP albeit much less abundant compared to ORF1p [35–37]. ORF1p and ORF2p strongly prefer binding their mRNA in a cis-acting manner [35,38,39]. Retrotransposition of LINE-1 involves reverse transcription of its RNA into DNA via a target-site priming mechanism that leads to integration of LINE-1 DNA into cellular DNA [34,40,41]. Zhao and colleagues reported that SAMHD1 diminishes LINE-1 reverse transcription by reducing the expression of ORF2 protein [27]. In this study, we report a new mechanism behind SAMHD1 inhibition of LINE-1. This mechanism involves the ability of SAMHD1 to induce the formation of large stress granules and thereby enhance the sequestration of LINE-1 RNP within these cytoplasmic foci. Depletion of G3BP1 or TIA1, key regulators of stress granule assembly [42,43], abrogates the ability of SAMHD1 to inhibit LINE-1 retrotransposition. Therefore, in addition to targeting ORF2 protein, SAMHD1 limits LINE-1 mobilization also by activating the stress granule pathway. This latter function may allow SAMHD1 to impact on diverse cellular events and restrict the replication of a broad range of microbes.

## Results

### SAMHD1 inhibits LINE-1 retrotransposition

To monitor LINE-1 activity, we utilized the CMV-L1-neo^RT^ reporter construct that has a neomycin resistance gene inserted into the 3’ UTR (untranslated region) of LINE-1 [44,45]. This neomycin resistance gene was such designed that it can only be expressed from the retrotransposed LINE-1 DNA. In agreement with the results reported by Zhao and colleagues [27], we also observed that depletion of endogenous SAMHD1 with short interference RNA (siRNA) increased LINE-1 retrotransposition in HeLa or HEK-293 cells by 3-fold (p<0.05) (S1A and S1B Fig). A similar increase in LINE-1 retrotransposition was observed when the endogenous SAMHD1 was depleted with SIV Vpx protein (S1C Fig). Furthermore, ectopically expressed SAMHD1 diminished LINE-1 retrotransposition by 5-fold (*p*<0.05) (S1D and S1E Fig). As a control for the specificity of SAMHD1 inhibition, a plasmid DNA named pcDNA3.1 (Invitrogen), which carries the neomycin resistance gene, was transfected into HeLa cells followed by G418 selection. No significant effect of SAMHD1 was observed on pcDNA3.1 induced G418 resistant colonies (S2A and S2B Fig). The results of cell proliferation assay further demonstrated that neither knockdown nor overexpression of SAMHD1 affected cell growth (S2C and S2D Fig). Similar to human SAMHD1, mouse SAMHD1 strongly inhibited LINE-1 by 5-fold (*p*<0.05) (S1F Fig).

SAMHD1 is a dNTP triphosphohydrolase and this enzymatic activity is essential for SAMHD1 to inhibit HIV-1 [4,5]. In order to test whether SAMHD1 also requires its triphosphohydrolase activity to inhibit LINE-1, we generated three mutants H233A, D311A and HDAA (H206A/D207A) that have been shown defective in dNTP hydrolysis [4,5]. The results of LINE-1 transposition assay showed that none of these three mutants inhibited LINE-1 (S1G Fig). We also measured the levels of wild type SAMHD1 and its mutants at day 3 and day 7 after transfection, and observed that the overall levels of wild type and mutated SAMHD1 moderately diminished from day 3 to day 7 except for mutant HDAA that virtually lost its expression at day 7 (S1H Fig). These data suggest that the triphosphohydrolase activity is required by SAMHD1 to inhibit LINE-1.

To determine which step of LINE-1 mobilization is affected by SAMHD1, we first tested whether SAMHD1 and its H233A mutant affect the expression of LINE-1 RNA or ORF1p, and no effect was observed (S3A Fig). We next performed PCR to measure the level of LINE-1 cDNA that represents the relative number of retrotransposition events as described in [46]. The forward primer binds to the spliced neomycin resistance gene sequence so that only the retrotransposed LINE-1 cDNA, but not the CMV-L1-neo^RT^ reporter DNA, is amplified. Consistent with the results of cell colony assays, results in S3B Fig show that SAMHD1 diminished LINE-1 retrotransposition by approximately 3-fold, whereas the H233A mutant did not exert any effect. This observation supports the report by Zhao and colleagues showing that SAMHD1 diminishes LINE-1 cDNA production in the LEAP (L1 element amplification protocol) RT assay by reducing ORF2p level [27].

### SAMHD1 causes sequestration of LINE-1 ORF1p into large cytoplasmic foci containing stress granule markers G3BP1 and TIA1

**Fig 1:**
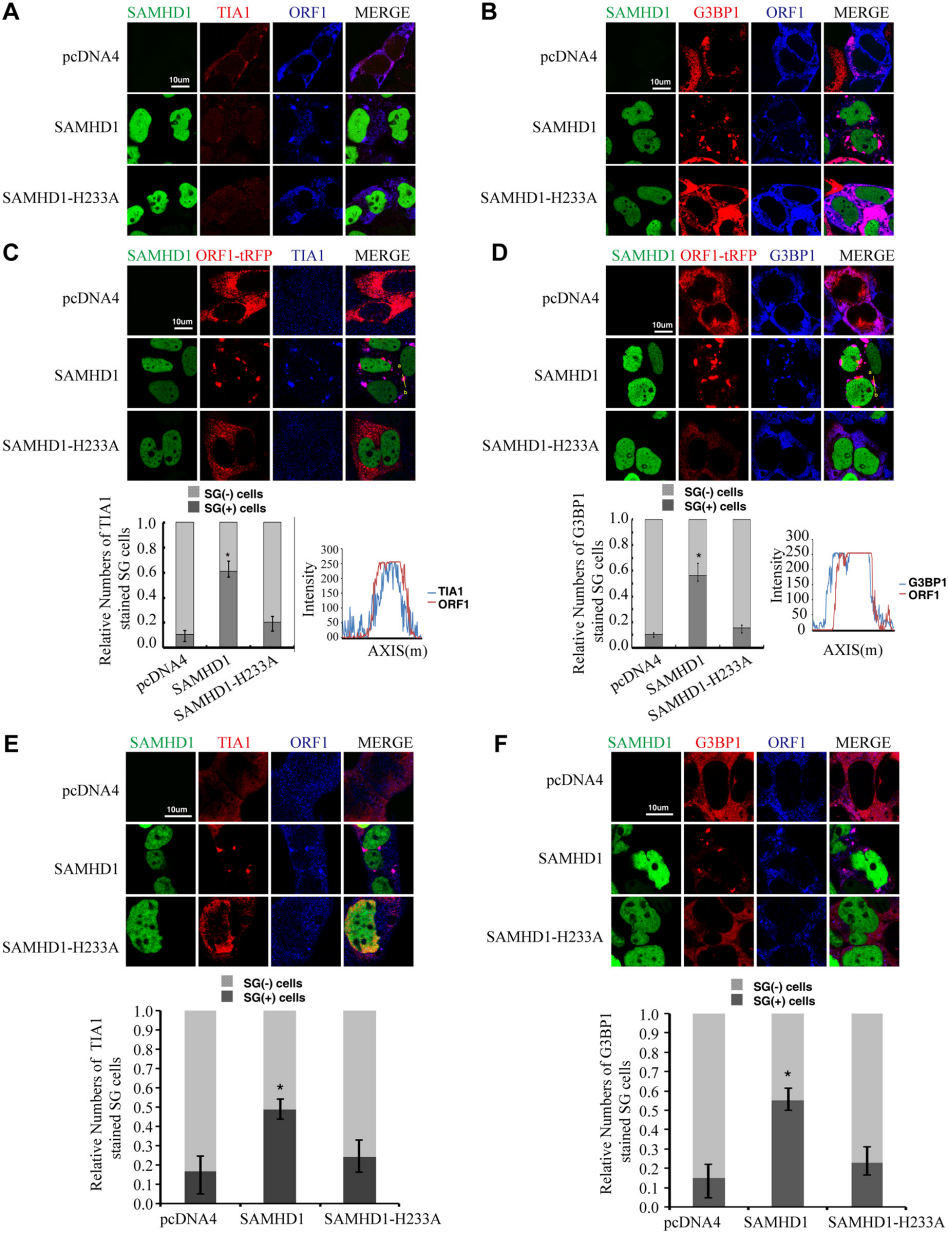
Ectopic expression of SAMHD1 leads to sequestration of LINE-1 ORF1p in stress granules. (A, B) The CMV-L1-neo^RT^ DNA and wild type EGFP-SAMHD1 or its H233A mutant DNA were transfected into HeLa cells. Endogenous TIA1 (shown in (A)) and G3BP1 (shown in (B)) were detected by indirect immunofluorescence staining. (C, D) The ORF1-tRFP DNA and wild type EGFP-SAMHD1 or its H233A mutant DNA were transfected into HeLa cells. Endogenous TIA1 (shown in (C)) and G3BP1 (shown in (D)) were detected by indirect immunofluorescence staining. A number of 200 cells were examined for each transfection to score the TIA1- or G3BP1-strained stress granule (SG) cells. The results are summarized in the bar graphs. Colocalization of TIA1 or G3BP1 with ORF1-tRFP was further analyzed with fluorescence intensity analysis software from LAS AF (Leica). (E, F) EGFP-SAMHD1 was expressed in HEK-293 cells. Cellular localizations of SAMHD1 and endogenous LINE-1 ORF1p were determined by immunostaining and confocal microscopy. Endogenous TIA1 (E), G3BP1 (F) and LINE-1 ORF1p were detected by indirect immunofluorescence staining. The number of SG-containing cells was calculated in more than 6 randomly chosen fields for each slide, 200 cells were examined in at least three independent transfections. The results are summarized in the bar graphs. Bars represents 10 μm. Statistical significance (Student's t test) was calculated, * indicates *p*< 0.05.

It is unclear how SAMHD1 might impair ORF2p expression. We suspected that SAMHD1 might operate via other mechanisms to inhibit LINE-1 retrotransposition. Indeed, results of immunofluorescence assays revealed an impact of SAMHD1 on the subcellular distribution of LINE-1 ORF1p (Fig 1). In HeLa cells that were transfected with LINE-1 DNA, ORF1p was dispersed in the majority of the cells (Fig 1A and 1B). ORF1p also formed cytoplasmic foci in about 10% of transfected cells, which is consistent with a previous report [47]. Upon overexpression of SAMHD1, ORF1p formed large cytoplasmic foci in about 55% of the cells examined (Fig 1A and 1B). When the stress granule marker proteins TIA1 or G3BP1 were visualized by immunofluorescence staining, the ORF1p foci in SAMHD1-expressing cells were TIA1- and G3BP1-positive (Fig 1A and 1B), which suggests that SAMHD1 causes sequestration of LINE-1 ORF1p in large stress granules that are over 1 μm in size. The same observation was made when only ORF1-tRFP fusion protein was co-expressed with SAMHD1. ORF1-tRFP formed large cytoplasmic foci in 60% of the cells that were transfected with SAMHD1 as compared to 10% of the cells that were transfected with a control plasmid (Fig 1C and 1D). In agreement with the inability of SAMHD1 mutant H233A to inhibit LINE-1 retrotransposition, H233A expression did not cause clustering of ORF1p (Fig 1A–1D). We further examined the effect of SAMHD1 on the endogenous ORF1p in HEK-293 cells. Without SAMHD1 overexpression, the endogenous ORF1p was dispersed in the majority of cells (Fig 1E and 1F). In contrast, SAMHD1 overexpression caused the association of endogenous ORF1p with large cytoplasmic foci that were positively stained for stress granule marker proteins in approximately 55% of the cells (Fig 1E and 1F).

**Fig 2:**
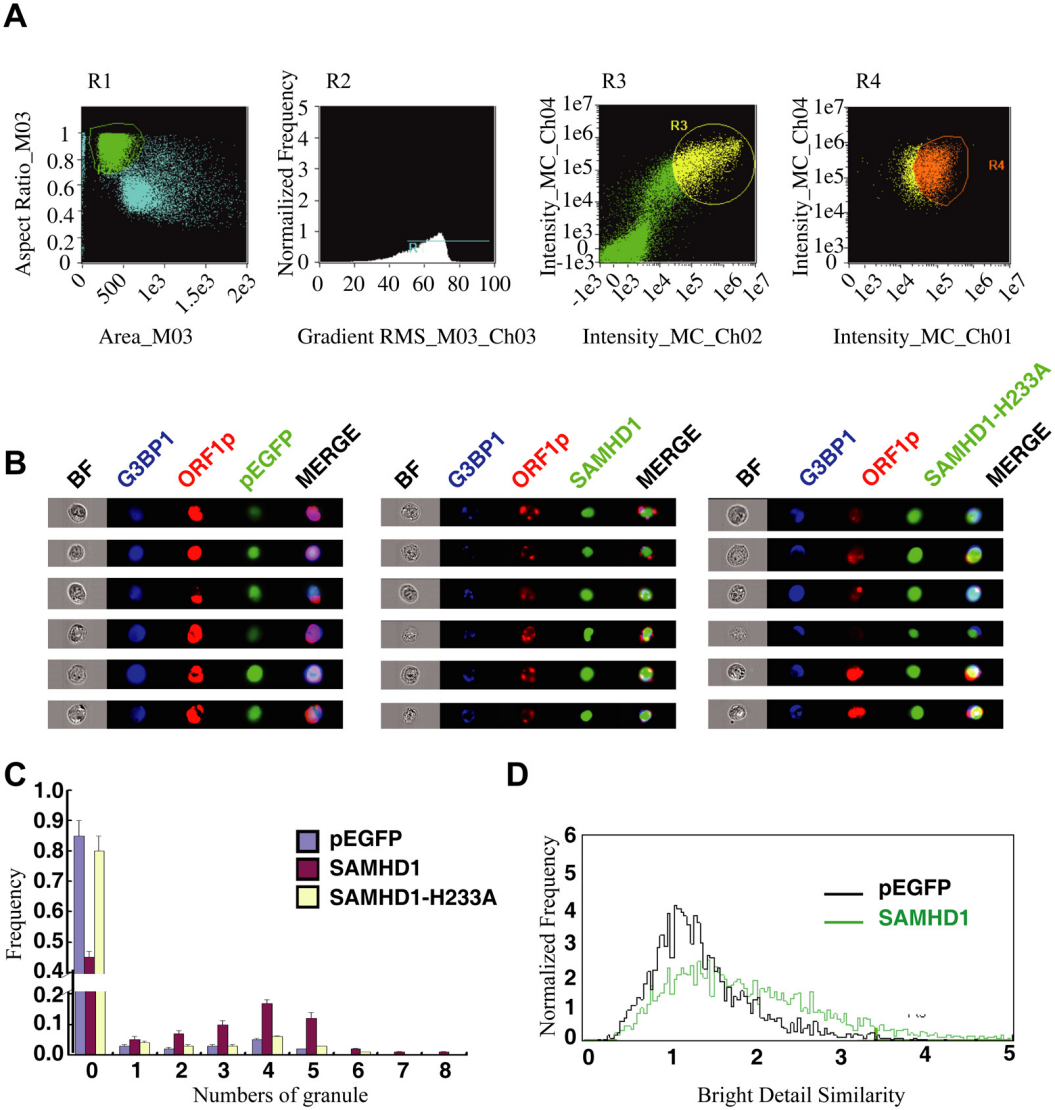
ImageStream flow cytometry was utilized to monitor the formation of stress granules and co-localization of SAMDH1 and ORF1p. HeLa cells were co-transfected with ORF1-tRFP and wild type EGFP-SAMHD1 DNA or the SAMHD1-H233A mutant DNA. Twenty-four hours post transfection, cells were stained with anti-G3BP1 antibody. Automated quantification of stress granule formation on gated cells was performed using the ImageStream technology. (A) Gating of cells. Single cells were first gated in R1, then cells in focus were selected in R2. EGFP and G3BP1 positive cells were selected in R3. tRFP, EGFP and G3BP1 positive cells were finally gated in R4 for imaging and further analysis. (B) Representative bright-field and fluorescence emission images for individual cells from each sample shown. The fluorescent granule inside each cell is clearly visible. (C) A number of 1x10^4^ cells were examined and the frequency of cells with different numbers of G3BP1-positive puncta is presented in the graph. (D) The degree of colocalization of G3BP1 and ORF1p was calculated as the bright detail similarity score. EGFP was utilized as the control. Cells with SAMHD1 overexpression have higher similarity values as a result of greater co-localization of G3BP1 and ORF1p.

We then used an automated method to quantify subcellular distribution of ORF1p by examining a large group of cells. The ImageStream system (Amnis) combines flow cytometry with intracellular imaging to calculate localization of fluorescent molecules [48]. A large cell population (10^4^ cells) was analyzed to score the SAMHD1-induced ORF1p foci and the co-localization of ORF1p with G3BP1. A 3-fold increase in the frequency of G3BP1/ORF1p granule formation was observed as a result of SAMHD1 overexpression, the H233A mutant did not exert any effect (Fig 2).

**Fig 3:**
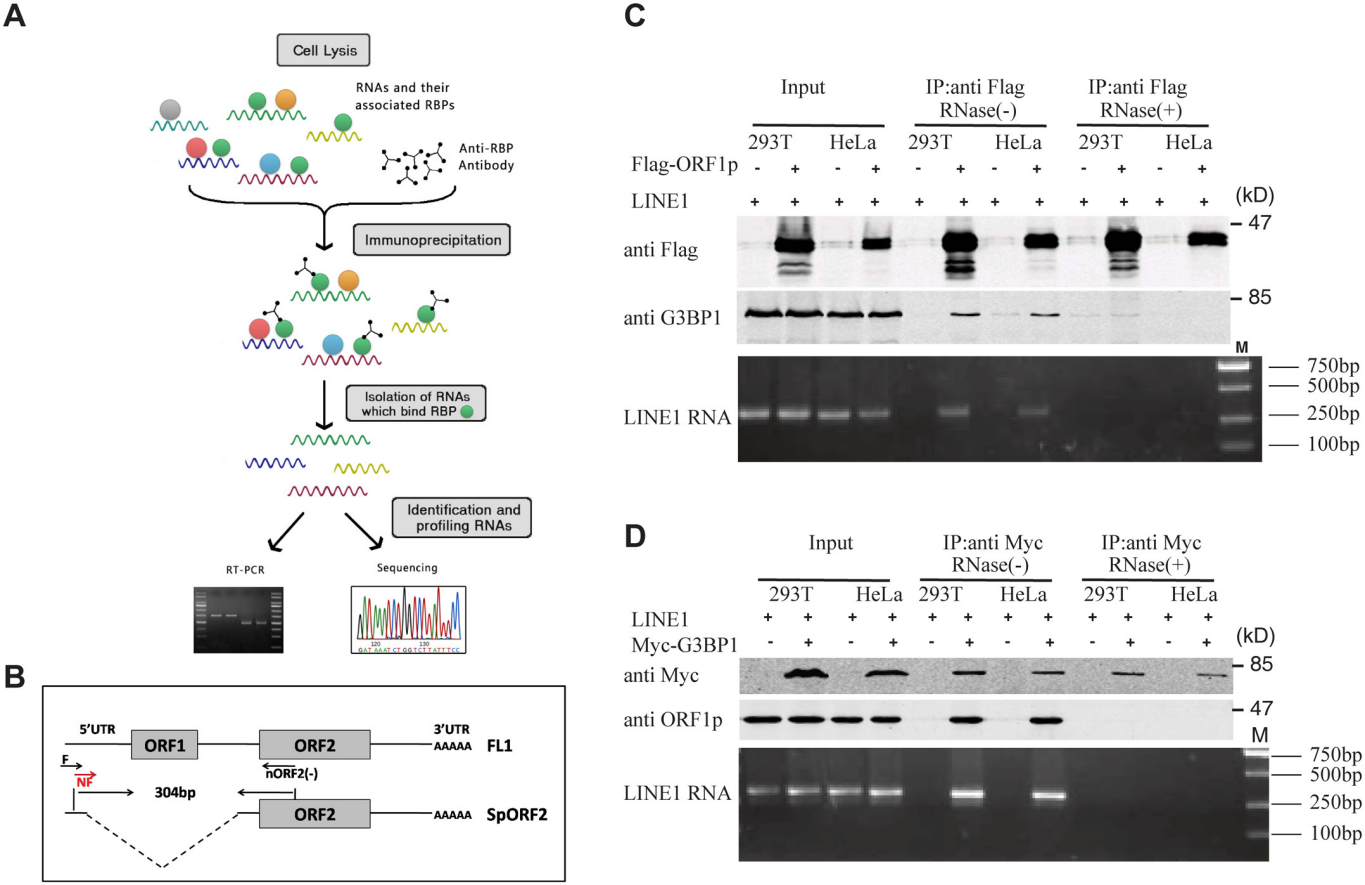
Association of LINE-1 RNP with stress granule marker G3BP1. (A) Schematic illustration of the immunoprecipitation/RT-PCR protocol. LINE-1 RNA that was co-immunoprecipitated (IP) was measured by nest RT-PCR. The PCR products were analyzed by gel electrophoresis followed by extraction from the gel and sequencing. (B) Depiction of the full-length LINE-1 RNA (FL1) and the spliced ORF2 transcript (SpORF2). Arrows indicate the position of the primers used in RT-PCR. This primer pair amplifies a 304 bp sequence of the SpORF2 RNA. (C) HeLa cells were transfected with Flag-ORF1p and CMV-L1-neo^RT^. Immunoprecipitation was performed with anti-Flag M2 antibodies to pull down Flag-ORF1p as well as ORF1p expressed from CMV-L1-neo^RT^. The immunoprecipitated materials were treated with or without RNase A during IP as indicated. Presence in the precipitated materials of ORF1p and G3BP1 was determined by western blotting. RT-PCR was performed to measure the levels of LINE-1 RNA in the precipitated samples. The PCR products were analyzed by gel electrophoresis. (D) HeLa cells were co-transfected with Myc-G3BP1 and CMV-L1-neo^RT^. G3BP1 was precipitated with anti-Myc antibody. The immunoprecipitated materials were treated with or without RNase A during IP as indicated. Co-immunoprecipitation of ORF1p and LINE-1 RNA was determined by western blotting and RT-PCR, respectively. The PCR products were analyzed by gel electrophoresis. The PCR products were also extracted from the gel and sequenced to confirm their being LINE-1 sequence.

**Fig 4:**
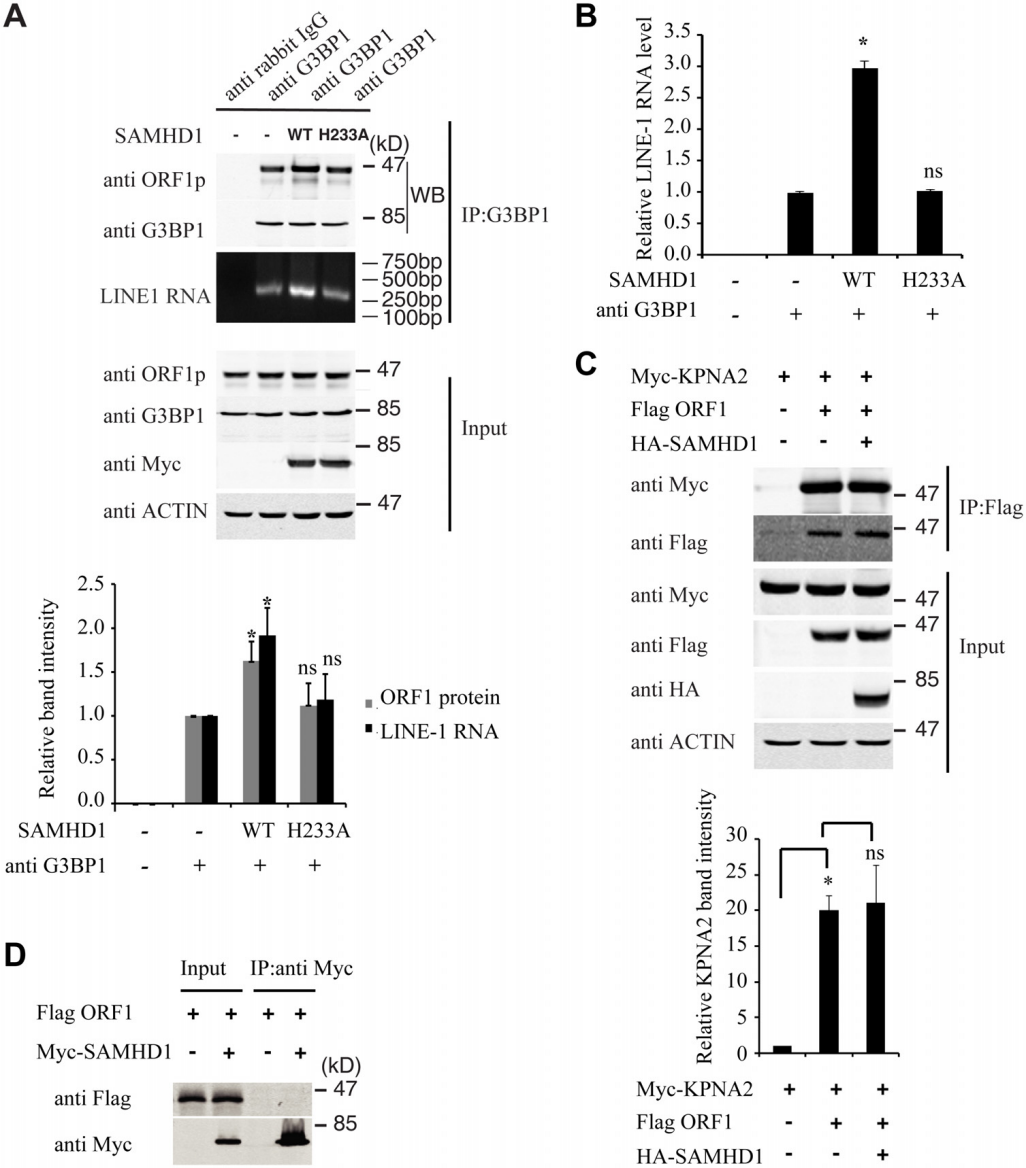
SAMHD1 increases LINE-1 RNP sequestration in stress granules. (A) HeLa cells were co-transfected with CMV-L1-neo^RT^ DNA and wild type Myc-SAMHD1 or SAMHD1-H233A mutant DNA. Endogenous G3BP1 was precipitated with anti-G3BP1 antibody. The anti-rabbit IgG was utilized as a control for non-specific binding. Co-immunoprecipitation of ORF1 and LINE-1 RNA was determined by western blotting and nest RT-PCR, respectively. Intensities of ORF1 bands (western blotting) and LINE-1 RNA bands (nest RT-PCR) were quantified using the ImageJ automated digitizing program (NIH). The results from three independent experiments are summarized in the bar graph. Levels of ORF1 and LINE-1 RNA in the control (SAMHD1 (-), G3BP1 antibody (+)) are arbitrarily set as 1. (B) Co-immunoprecipitation of LINE-1 RNA was determined by real-time quantitative PCR as described in Materials and Methods. The results from three independent experiments are summarized in the bar graph. Levels of ORF1 and LINE-1 RNA in the control (SAMHD1 (-), G3BP1 antibody (+)) are arbitrarily set as 1. (C) HeLa cells were co-transfected with Myc-KPNA2, Flag-ORF1 with or without HA-SAMDH1. Immunoprecipitation was performed with anti-Flag M2 gel. Presence of Myc-KPNA2 in the precipitated materials was determined by western blotting. Intensities of the KPNA2 bands were quantified using the ImageJ automated digitizing program (NIH), the results from three independent experiments are summarized in the bar graph. Level of KPNA2 in the control cells is arbitrarily set as 1. (D) SAMHD1 is not associated with ORF1. HeLa cells were co-transfected with Myc-SAMHD1 DNA and ORF1-Flag DNA. Myc-SAMHD1 was immunoprecipitated with anti-Myc antibody. The presence of ORF1 in the precipitated materials was examined by western blotting. * indicates p<0.05, ns denotes “not significant”.

Association of LINE-1 RNP with stress granules has been previously reported [36,37,49]. Results of our co-immunoprecipitation and RT-PCR assays further confirmed the association of stress granule marker protein G3BP1 with LINE-1 ORF1p and LINE-1 RNA in an RNase-sensitive manner (Fig 3). In order to avoid the contamination of RT-PCR by the transfected LINE-1 DNA, we designed primers to amplify a spliced form of LINE-1 RNA called SpORF2 that is devoid of the ORF1p sequence (Fig 3B) [50]. We further sequenced the RT-PCR products and confirmed that the amplified sequence resulted from a splicing event that connects LINE-1 nucleotide positions 97 and 1837 as previously reported [51]. We next tested the effect of SAMHD1 on the association of G3BP1 with LINE-1 ORF1p and RNA. The results of Fig 4A and 4B show that SAMHD1, not its H233A mutant, increased the interaction of G3BP1 with LINE-1 ORF1p and RNA by nearly 3-fold. This effect of SAMHD1 appears to be specific, since the interaction of another cellular protein called KPNA2 with ORF1p, which has been reported to take place in an RNA-dependent manner by Goodier and colleagues [52], was not affected by SAMHD1 overexpression, as shown by the results of co-immunoprecipitation assay (Fig 4C) [52]. We did not detect any interaction between SAMHD1 and ORF1p (Fig 4D), which indicates that SAMHD1 promotes sequestration of LINE-1 ORF1p to stress granules by an indirect mechanism. Together, these data suggest that the effect of SAMHD1 on cytoplasmic sequestration of LINE-1 RNP stems from two related events. First, SAMHD1 stimulates the formation of stress granules where LINE-1 ORF1p is sequestered as shown in Fig 1. Second, SAMHD1 enhances the intrinsic association of G3BP1 with LINE-1 ORF1p and RNA, and thus further promotes sequestration of LINE-1 RNP to stress granules (Fig 4A and 4B).

### Stress granule pathway restricts LINE-1 activity

**Fig 5:**
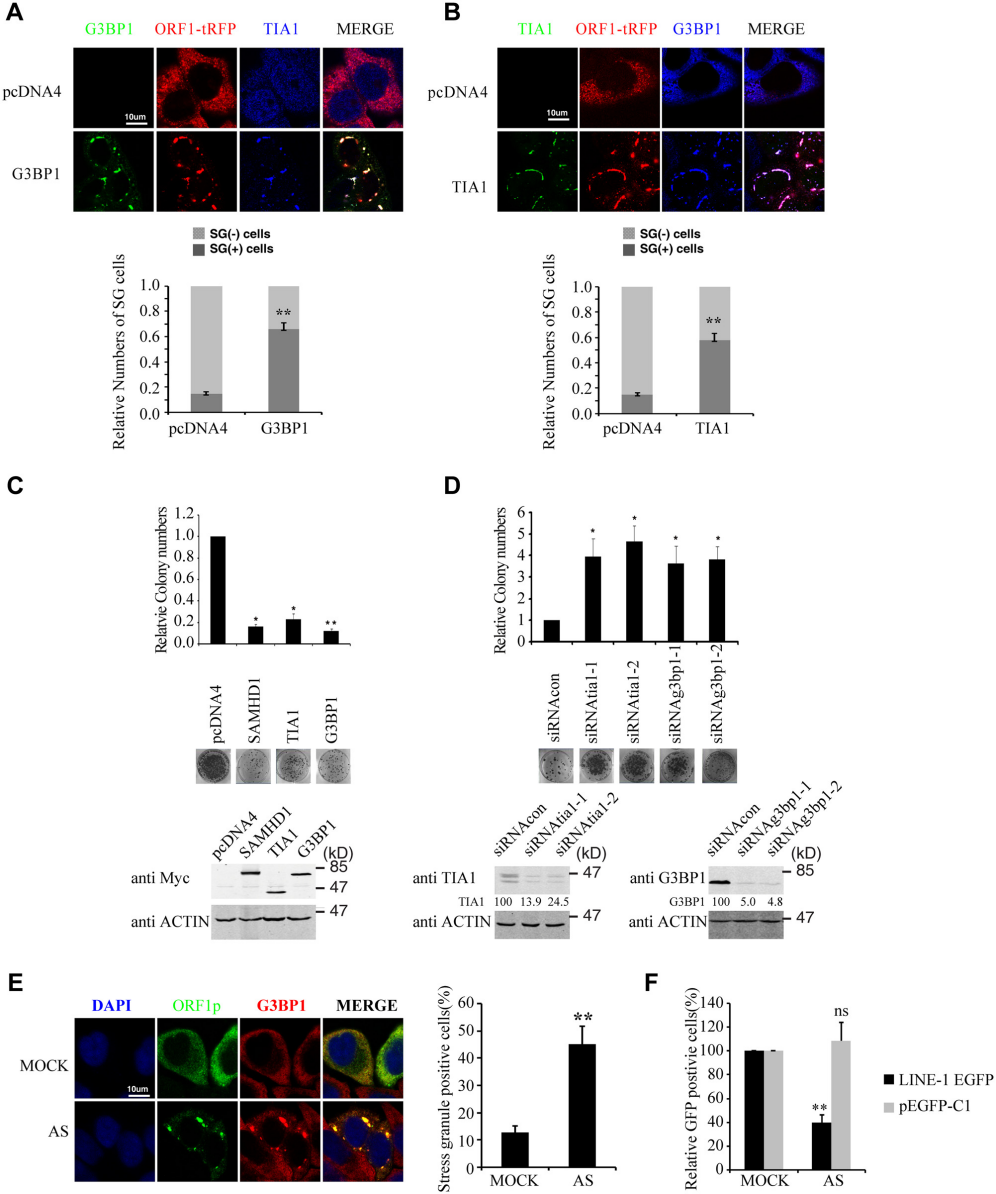
Stress granule pathway restricts LINE-1 retrotransposition. (A) The ORF1-tRFP DNA and Myc-G3BP1 DNA were transfected into HeLa cells. Cells were stained with mouse anti-Myc antibody and rabbit anti-TIA1 antibody. (B) The ORF1-tRFP DNA and Myc-TIA1 DNA were transfected into HeLa cells. Cells were stained with mouse anti-Myc antibody and rabbit anti-G3BP1 antibody. The number of SG-containing cells was calculated in more than 6 randomly chosen fields for each slide, and 200 cells were examined in at least three independent transfections. The results are summarized in the bar graphs. (C) HeLa cells were transfected with CMV-L1-neo^RT^ DNA together with SAMHD1, TIA1 or G3BP1 DNA. Neomycin-resistant cell colonies were scored and results of three independent experiments are presented in the bar graph. Number of neomycin-resistant colonies with control vector is arbitrarily set as 1. Results of three independent experiments are shown in the bar graph. Images of a representative colony assay are presented. Ectopic expression of Myc-SAMHD1, Myc-TIA1 or Myc-G3BP1 was examined by western blotting. (D) Knockdown TIA1 or G3BP1 increases LINE-1 activity. HeLa cells were treated with siRNA targeting either TIA1 or G3BP1 prior to transfection with LINE-1 reporter DNA CMV-L1-neo^RT^. Neomycin-resistant cell colonies were scored and results of three independent experiments are presented in the bar graph. Number of neo-resistant colonies with control siRNA is arbitrarily set as 1. The knockdown of TIA1 or G3BP1 was examined by western blotting. Intensities of TIA1 or G3BP1 bands were determined using the ImageJ program. The results were used to calculate the knockdown efficiency as indicated under the western blots. (E) The CMV-L1-neo^RT^ DNA was transfected into HeLa cells. 6 hours post transfection, cells were cultured in the presence of 1 μM arsenite (AS) for 48h, then fixed and processed for visualization of G3BP1 and ORF1p using fluorescence microscopy. The number of SG-containing cells was calculated in more than 6 randomly chosen fields for each slide, and 200 cells were examined in at least three independent transfections. The results are summarized in the bar graphs in the right panel. Bars represents 10 μm. (F) The LINE-1 EGFP reporter DNA was transfected into 293T cells. Six hours post transfection, cells were treated with 1 μM arsenite for 48 h. GFP-positive cells were scored by flow cytometry at day 5. As a control, cells were also transfected with the pEGFP-C1 vector DNA that constitutively expresses EGFP. Results are normalized to control (without arsenite) and summarized in the bar graphs (n = 3). * indicates *p*< 0.05, ** represents *p*< 0.01.

If increasing sequestration of LINE-1 RNP to stress granules represents one mechanism underpinning the inhibitory activity of SAMHD1, then stress granule pathway itself may have a role in controlling LINE-1 retrotransposition. To test this, we first overexpressed G3BP1 or TIA1 in HeLa cells, which causes stress granule formation as previously reported (Fig 5A and 5B)[53,54]. When LINE-1 retrotransposition was measured, a 5-fold reduction was observed under overexpression of either G3BP1 or TIA1 (Fig 5C). As a control, overexpression of these two proteins neither affected the formation of G418-resistant colonies as a result of transfection of pcDNA3.1 nor cell proliferation (S2B and S2D Fig). This inhibitory activity of G3BP1 or TIA1 was corroborated by a 4-fold increase in LINE-1 retrotransposition when the endogenous G3BP1 or TIA1 was knocked down (Fig 5D). In order to further demonstrate the inhibitory effect of stress granules on LINE-1 activity, we treated cells with arsenite (AS) that potently induced the formation of stress granule (Fig 5E) without any discernible effect on cell proliferation in short term (S2E Fig). When LINE-1 retrotransposition in arsenite-treated cells was determined, a 3-fold reduction was observed (Fig 5F). Collectively, these data suggest that stress granule pathway contributes to cellular control of LINE-1 activity.

### The inhibition of LINE-1 by SAMHD1 depends on the stress granule pathway

**Fig 6:**
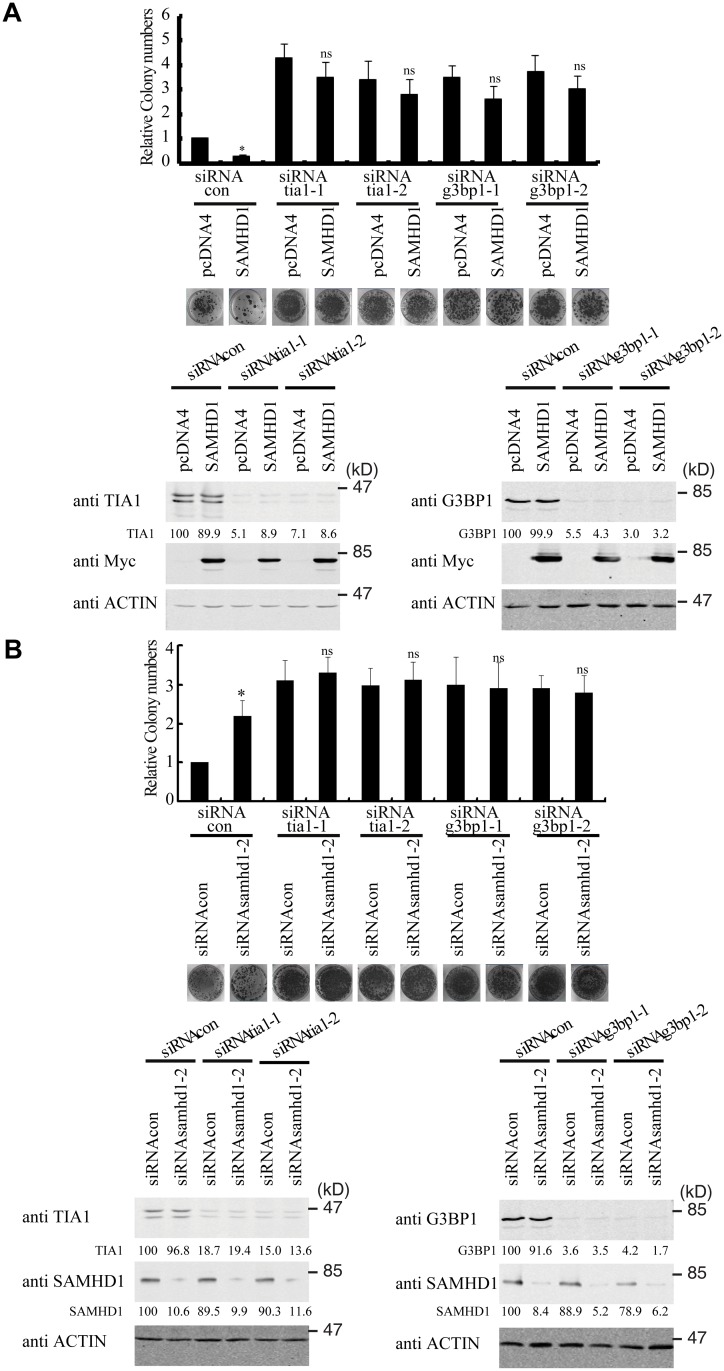
TIA1 and G3BP1 are required for SAMHD1 to inhibit LINE-1 retrotransposition. (A) Knockdown of TIA1 or G3BP1 prevents the SAMHD1 from inhibiting LINE-1. HeLa cells were transfected with siRNA targeting TIA1 or G3BP1 prior to co-transfection with Myc-SAMHD1 and CMV-L1-neo^RT^. Neomycin-resistant colonies were scored and results of three independent experiments are shown in the bar graph. Levels of TIA1, G3BP1 and Myc-SAMHD1 were determined by western blotting. The knockdown efficiency of TIA1 and G3BP1 was calculated on the basis of the intensities of TIA1 or G3BP1 bands in the western blots. (B) Endogenous SAMHD1 loses inhibition of LINE-1 upon depletion of TIA1 or G3BP1. HeLa cells were treated with siRNAs targeting TIA1, G3BP1 or SAMHD1, followed by transfection with CMV-L1-neo^RT^ DNA. Number of neomycin-resistant colonies was determined and shown in the bar graph. The number of neomycin-resistant colonies with control siRNA is arbitrarily set as 1. Levels of endogenous TIA1, G3BP1 and SAMHD1 were examined by western blotting. The knockdown efficiency was calculated on the basis of the protein band intensities as determined using the ImageJ program. * indicates *p*< 0.05, ns denotes “not significant”.

**Fig 7:**
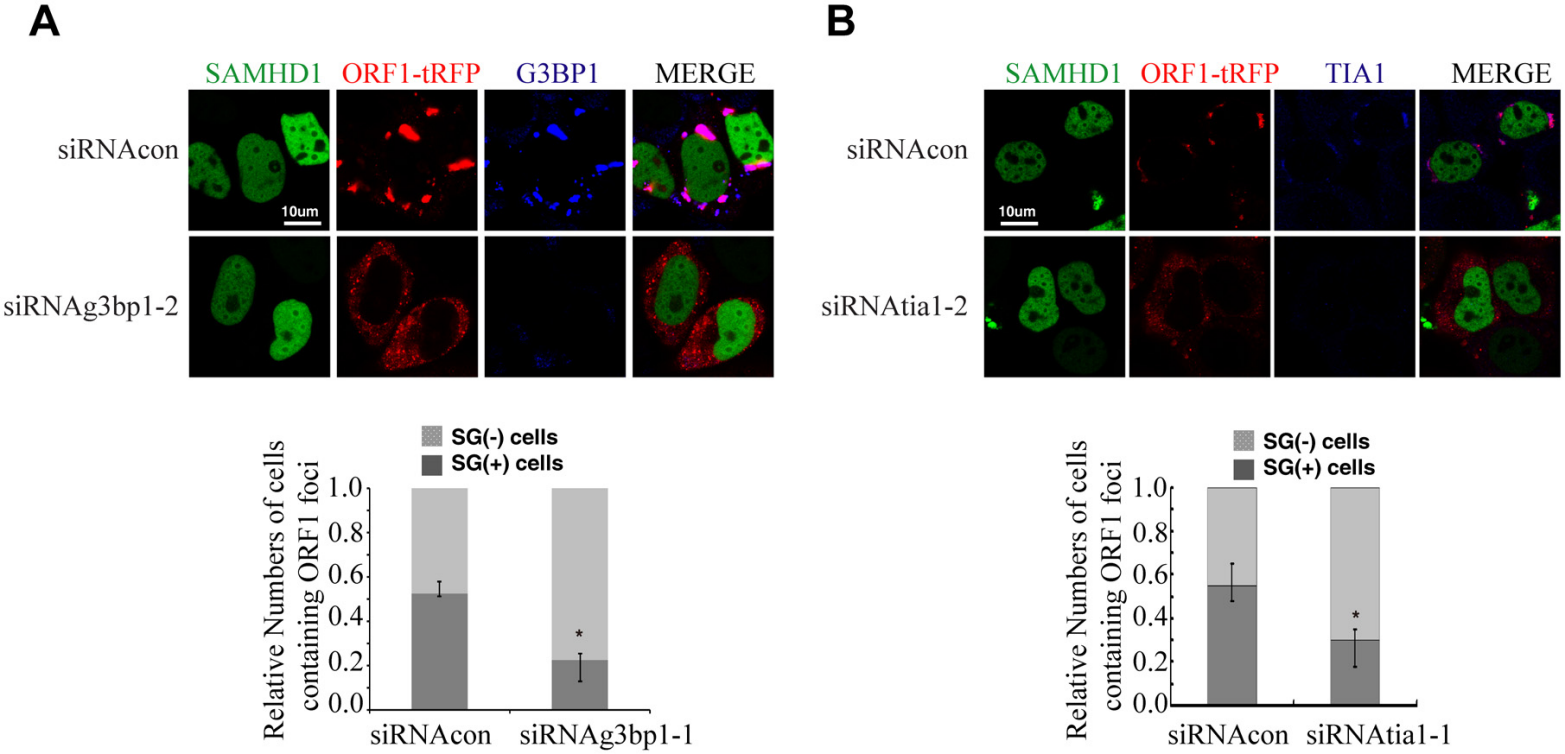
TIA1 and G3BP1 are required for SAMHD1 to form large ORF1p foci. HeLa cells were transfected with siRNA oligos targeting G3BP1 (A) or TIA1 (B) and plasmid DNA encoding EGFP-SAMHD1 and ORF1-tRFP. Twenty-four hours post transfection, cells were stained with rabbit anti-G3BP1 antibody or anti-TIA1 antibody (blue), Alexa Fluor 647 labeled Goat anti-Rabbit antibody were used as secondary antibodies. Relative number of G3BP1 or TIA1 stained cells means that the percentages of SG-containing cells were calculated in more than 6 randomly chosen fields for each slide with examining 200 cells. The results are summarized in the bar graphs. Bars represents 10 μm. * indicates *p*< 0.05.

We next tested whether SAMHD1 inhibits LINE-1 through enhancing this intrinsic inhibitory function of stress granule pathway. To this end, we blocked stress granule pathway by depleting endogenous G3BP1 or TIA1, and examined whether SAMHD1 could still inhibit LINE-1. As expected, knockdown of TIA1 or G3BP1 prevented stress granule formation under arsenic treatment or SAMHD1 overexpression (S4 Fig). We first overexpressed SAMHD1 and observed a 4-fold decrease in LINE-1 retrotransposition in cells that were treated with control siRNA (Fig 6A). This inhibition by SAMHD1 was virtually lost in cells that were depleted of either TIA1 or G3BP1 (Fig 6A). The marginal reduction of LINE-1 activity caused by SAMHD1 in these knockdown cells might result from the residual amounts of TIA1 or G3BP1 following siRNA treatment. Next, we knocked down endogenous SAMHD1, which led to a 2-fold increase in LINE-1 retrotransposition (Fig 6B). Knockdown of either TIA1 or G3BP1 increased LINE-1 retrotransposition by 3-fold (Fig 6B). Importantly, in these TIA1 or G3BP1 depleted cells, SAMHD1 knockdown did not lead to a further increase in LINE-1 retrotransposition (Fig 6B). In support of these functional data, depletion of TIA1 or G3BP1 led to the loss of large ORF1p foci in SAMHD1-expressing cells, although in approximately 20% of the cells ORF1 proteins still tend to form much smaller puncta in the background of the dispersed ORF1p (Fig 7). Our data do not exclude the possibility that knockdown of TIA1 or G3BP1 may distribute ORF1p from stress granules to p-bodies. Collectively, these suggest that SAMHD1 inhibition of LINE-1 depends on the function of stress granule pathway. We cannot rule out the possibility that knockdown of either TIA1 or G3BP1 may have led to the maximum activation of LINE-1 and therefore knockdown of SAMHD1 no longer produces any further increase.

### SAMHD1 promotes the formation of large stress granules

**Fig 8:**
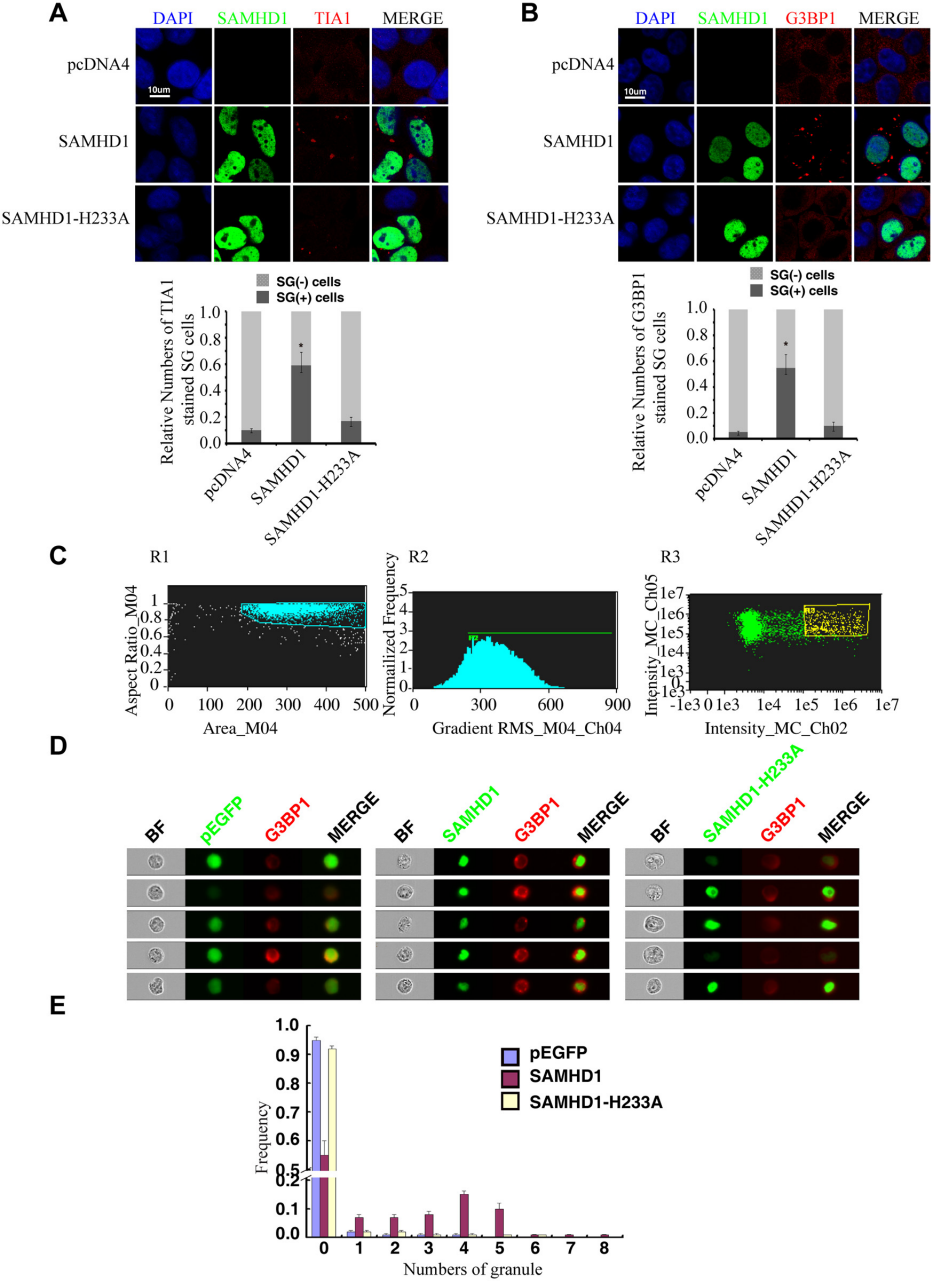
SAMHD1 overexpression induces stress granules formation. (A, B) HeLa cells were transfected with pcDNA4, EGFP-SAMHD1 or EGFP-SAMHD1-H233A. Cells were collected at 24h post transfection and immunostained for cellular TIA1 (A) or G3BP1 (B). The secondary antibodies used were Alexa Fluor 594-labeled goat anti-Rabbit antibody (red). The proportion of cells with SGs (%) is shown in the bar graphs. The data are shown as the average of three independent experiments. (C, D, E). ImageStream flow cytometry was utilized to monitor the formation of stress granules in the presence of SAMDH1. HeLa cells were transfected with the wild type EGFP-SAMHD1 DNA or the H233A mutant. 48h post transfection, cells were stained with anti-G3BP1 antibody. (C) Cell populations were first gated for single cells (R1) in focus (R2). EGFP and G3BP1 positive cells were then gated in R3 for further analysis. (D) Representative bright-field and fluorescence emission images for individual cells from each sample shown. The fluorescent granule inside each cell is clearly visible. (E) The frequency of cells with different numbers of G3BP1-positive puncta was calculated in a population of 1x10^4^ cells. The results are shown in the graph. Bars represent 10 μm.

We next tested whether SAMHD1 itself can modulate the stress granule pathway. In support of this possibility, overexpressing SAMHD1 alone, in the absence of LINE-1 protein and RNA expression, induced the formation of large stress granules containing TIA1 and G3BP1 (Fig 8A and 8B). The H233A mutant, which is unable to inhibit LINE-1, did not markedly affect the distribution of TIA1 or G3BP1 (Fig 8A and 8B). These results were further supported by the data of ImageStream flow cytometry showing that wild type SAMHD1, but not the H233A mutant, markedly increased the number of cells having large stress granules (Fig 8C–8E).

**Fig 9:**
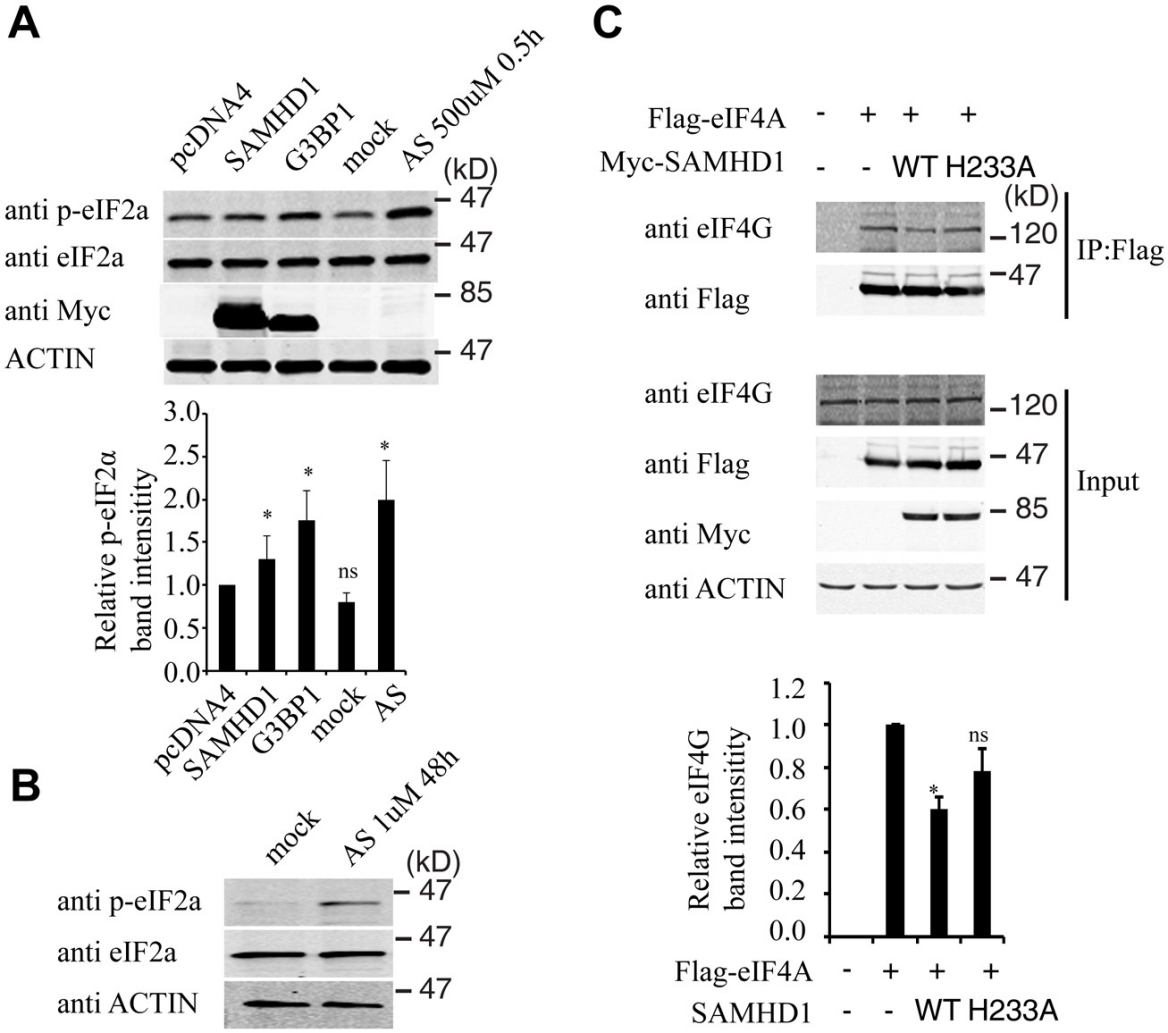
SAMHD1 modulates the phosphorylation of eIF2α and the interaction of eIF4G and eIF4A. (A) Effect of SAMHD1 on eIF2α phosphorylation. HEK-293T cells were transfected with Myc-SAMHD1 or Myc-G3BP1 cDNA. Cells were also treated with arsenite (AS, 500 μM) for 0.5 hour that is known to induce eIF2α phosphorylation. 48h post transfection, cells were treated with Calyculin A (0.1 μM for 30 minutes prior to harvesting cells) to inhibit phosphatases. Western blotting was performed to measure phosphorylation of eIF2α using antibodies as indicated for each gel panel. Intensities of the phosphorylated eIF2α bands were quantified and the results of three independent transfections are shown in the bar graph. pcDNA4 is an empty vector and was used as a transfection control. (B) Lysates were prepared from HeLa cells treated with 1 μM arsenite for 48h. Immunoblotting was performed to analyze phosphorylation of eIF2α (p-eIF2α). (C) Effect of SAMHD1 and SAMHD1-H233A on the association of eIF4A and eIF4G. HeLa cells were transfected with Flag-eIF4A together with Myc-SAMHD1 or Myc-SAMHD1-H233A DNA. Flag-eIF4A was immunoprecipitated with anti-Flag M2 antibodies. Western blots were performed with rabbit anti-Flag, rabbit anti-eIF4G, rabbit anti-Myc (to detect Myc-SAMHD1), or mouse anti-actin antibodies. Intensities of the eIF4G bands were quantified using the ImageJ automated digitizing program (NIH). The bar graph shows the results of three independent transfection experiments. * indicates *p* < 0.05, ns denotes “not significant”.

Stress granule formation is often associated with translation arrest concomitant with phosphorylation of eIF2α [54–56]. A 30% increase in eIF2α phosphorylation was detected under SAMHD1 overexpression, albeit not as strong as the 2-fold increase that was induced by G3BP1 or arsenite treatment (Fig 9A). In agreement with their enhancing eIF2α phosphorylation, both G3BP1 overexpression (Fig 5C) and arsenite treatment (Fig 5F) reduced LINE-1 retrotransposition. In order to prolong the treatment with arsenite in the LINE-1 retrotransposition assay without causing cell toxicity, a low concentration of arsenite (1 μM) was chosen to treat LINE-1 DNA transfected cells for 48 hours. A strong increase in eIF2α phosphorylation was detected under this treatment condition (Fig 9B). We also explored whether SAMHD1 perturbs translation by mechanisms other than eIF2α phosphorylation. To this end, we examined the effect of SAMHD1 on the assembly of the eIF4F complex in which eIF4G serves as the scaffold by interacting with eIF4E and eIF4A [57]. The results of co-immunoprecipitation experiments showed a 40% decrease in the association of eIF4G with eIF4A in SAMHD1-expressing cells as compared to the control cells and no significant effect from the H233A mutant in this regard (Fig 9C). A precedent of this inhibitory effect of SAMHD1 is the zinc finger antiviral protein (ZAP) that has been reported to disrupt the eIF4A and eIF4G complex by interacting with eIF4A [58]. It remains to be elucidated how SAMHD1 modulates eIF2α phosphorylation as well as the association between eIF4A and eIF4G. Together, these data suggest that SAMHD1 might promote stress granule formation by inducing translation arrest.

### Endogenous SAMHD1 is involved in stress granule assembly

**Fig 10:**
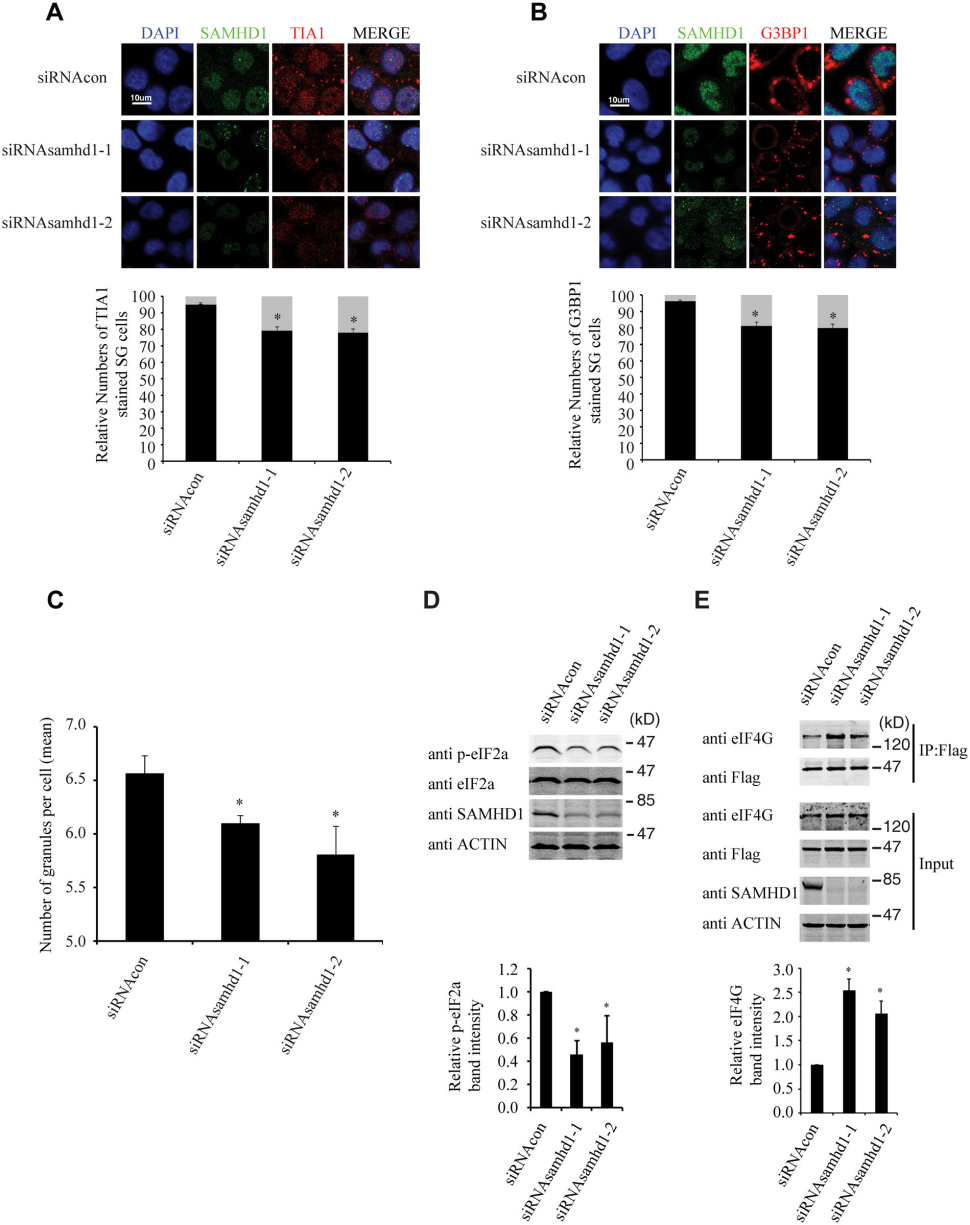
Knockdown of SAMHD1 diminishes the formation of stress granules. (A, B) HeLa cells were transfected with siRNA oligos targeting SAMHD1. 72h post transfection, cells were treated with arsenite (500 μM) for 30 min to induce stress granules. Cells were then stained with mouse anti-SAMHD1 antibody and rabbit anti-TIA1 (A) or anti-G3BP1 (B) antibody. Alexa Fluor 488 labeled goat anti-rabbit antibody or Alexa Fluor 594 labeled goat anti-rabbit antibody was used as the secondary antibody. (C) The Operetta High-Content Screen system (PerkinElmer) was utilized to score the number of G3BP1-positive stress granules in HeLa cells that were transfected with siRNA targeting SAMHD1 or control siRNA. Cells were treated with arsenite (500 μM) for 30 min to induce stress granules. The average numbers of stress granules in the control or SAMHD1-knockdown cells are shown in the bar graph. (D) The endogenous SAMHD1 in HeLa cells was first knocked down with siRNA, followed by treatment with arsenite (500 μM) for 30 min. Western blotting was performed to measure phosphorylation of eIF2α using specific antibodies as indicated for each panel. Intensities of the phosphorylated eIF2α bands were quantified using the ImageJ automated digitizing program (NIH) and results are summarized in the bar graph. Results shown are the average of three independent experiments. (E) HeLa cells were transfected with siRNA targeting SAMHD1 prior to transfection with Flag-eIF4A. Flag-eIF4A was immunoprecipitated with mouse anti-Flag antibody and probed with rabbit anti-Flag, rabbit anti-eIF4G, mouse anti-SAMHD1 or mouse anti-actin antibodies. Intensities of the eIF4G bands were quantified and the results of three independent transfection experiments are summarized in the bar graph. Bars represents 10 μm. * denotes p<0.05.

We further investigated whether the endogenous SAMHD1 has a role in stress granule formation. The results of immunofluorescence and confocal imaging showed that knockdown of SAMHD1 moderately diminished stress granule formation by 20% under arsenite treatment (Fig 10A and 10B). This observation was confirmed by the data obtained with the high-throughput screen platform (Operetta) by examining a large number of cells, which showed that the mean number of stress granules per cell in control cells was 6.6, and decreased to 5.7 with knockdown of SAMHD1 (Fig 10C). As opposed to the effects of overexpressed SAMHD1 on eIF2α phosphorylation and the eIF4G and eIF4A interaction that were shown in Fig 9, SAMHD1 knockdown decreased eIF2α phosphorylation and enhanced eIF4G and eIF4A interaction (Fig 10D and 10E). Together, these data support a role of the endogenous SAMHD1 in stress granule assembly.

## Discussion

Results of this study suggest that SAMHD1 is able to enhance stress granule formation, and by so doing, promotes sequestration of LINE-1 RNP in stress granules and thus limits LINE-1 retrotransposition (S5 Fig). This mechanism of SAMHD1 inhibition stems from the intrinsic function of the stress granule pathway to control LINE-1 activity. Localization of LINE-1 RNP (RNA, ORF1p and ORF2p) to stress granules has already been reported by a couple of groups [36,37,49]. This co-localization was observed in 10% to 15% of the cells in our experiments, which might be a result of the different sources of anti-ORF1p antibodies utilized in different studies. Nonetheless, the results of our immunoprecipitation and RT-PCR experiments do support a strong association of LINE-1 RNP with stress granule marker protein G3BP1 (Fig 3). More importantly, depletion of either G3BP1 or TIA1 increases in LINE-1 retrotransposition, which argues for an intrinsic role of stress granule pathway proteins in controlling LINE-1 retrotransposition. In agreement with this conclusion, overexpression of G3BP1 or TIA1, which elevates stress granule formation, strongly suppresses LINE-1 mobility. It is foreseeable that not only SAMHD1, but also other conditions that enhance stress granule formation, should stimulate cytoplasmic sequestration of LINE-1 RNP and dampen LINE-1 retrotransposition.

It is not completely clear how SAMHD1, as a nuclear protein, enhances stress granule formation in the cytoplasm. SAMHD1 may do so by modulating the phosphorylation of eIF2α and/or the interaction of eIF4G and eIF4A by unknown mechanisms (Figs 9 and 10). The role of eIF2α in LINE-1 retrotransposition has not been determined, although ORF1p distribution does not differ significantly between the wild type mouse embryonic fibroblasts (MEFs) and KO MEFs that lack eIF2α kinases PERK, PKR or GCN2 [49]. In any event, the resulted suppression of translation not only triggers the downstream stress responses but may also contribute to the reduction in LINE-1 ORF2p level as reported by Zhao and colleagues [27].

A number of cellular mechanisms have been reported to control LINE-1 activity mainly due to the fact that there still exist over 100 copies of active LINE-1 in human genome and retrotransposition of these LINE-1s causes mutations and diseases (reviewed in [28]). DNA methylation appears to play an important role in controlling LINEs during development [59–61]. The pi-RNA contributes to limit LINE retrotransposition in germ lines [62,63]. In addition, multiple cellular proteins have been shown to restrict LINE-1 activity, these include APOBEC3 proteins [64–68], MOV10 [69–71], SAMHD1 [27] and others. Using different approaches, two groups independently discovered that the zinc-finger antiviral protein (ZAP) inhibits LINE-1 retrotransposition by associating with LINE-1 RNP and cytoplasmic stress granules[72,73]. Together with other studies [36,37,49], our data further support a role of the stress granule pathway in controlling LINE-1 retrotransposition through sequestering LINE-1 RNP within the cytoplasm, which unravels a new layer of cellular restriction of LINE-1 activity.

Our data suggest a role of the triphosphohydrolase activity in SAMHD1 inhibition of LINE-1, which does not fully agree with the results shown by Zhao and colleagues [27]. We tested three SAMHD1 mutants H233A, D311A and HDAA that each had the key residues changed in SAMHD1 catalytic site. All mutants lost the ability to inhibit LINE-1 retrotransposition (S1G Fig). Zhao et al tested the 311A mutant in their study and observed substantial suppression of LINE-1 activity by this mutant although not as strong as the wild type SAMHD1 [27]. This discrepancy might be due to the fact that the two groups utilized different LINE-1 reporter system. We had used the colony-based CMV-L1-neo^RT^ reporter, whereas Zhao et al employed the EGFP-based LINE-1 reporter system [27,74]. Further studies are warranted to further investigate the involvement of the triphosphohydrolase activity in SAMHD1 inhibition of LINE-1.

It is not a surprise that stress granules limit LINE-1 mobility given the known interactions between viruses and stress granules. Many viruses have evolved means to manipulate the formation of stress granules or even co-opt stress granule components to promote their replication [75,76]. This is not only due to the key role of stress granules in RNA metabolism, but also results from the role of stress granules as a platform to orchestrate the antiviral innate immune response [77]. Results of our study support the scenario that stress granules serve as a defense mechanism of curtailing LINE-1 mobilization. It can be appreciated that factors or stimuli that promote stress granule formation are expected to affect LINE-1 retrotransposition. The ability to promote the formation of large stress granules may enable SAMHD1 not only to inhibit LINE-1 but also many viruses and therefore endow SAMHD1 with a new antiviral mechanism in addition to blocking viral DNA synthesis through lowering cellular dNTP pool. Along this line, more RNA viruses may become the target of SAMHD1 restriction.

The ability to inhibit LINE-1 suggests that SAMHD1 may affect the activity of other retrotransposons including endogenous retroviruses. Indeed, studies have shown that the non-autonomous retroelements Alu and SVA are inhibited by SAMHD1 [27]. This role of SAMHD1 in controlling the activity of endogenous retroelements, especially in macrophages and dendritic cells that are major interferon producer cells and express high level of SAMHD1, is expected to prevent the cytoplasmic accumulation of DNA and thus avoid the abnormal production of interferon and inflammatory cytokines. This function of SAMHD1 at least partially explains the association of its nonfunctional mutations with AGS that is featured by persistent interferon production reminiscent of congenital infection [2].

## Materials and Methods

### Plasmids and antibodies

The CMV-L1-neo^RT^ DNA reports LINE-1 retrotransposition by producing neomycin (G418)-resistance cell colonies and was a kind gift of Thierry Heidmann and Daniel Stetson [44]. This reporter construct is a derivative of the L1.2A clone (accession number M80343)[78]. The LINE-1 EGFP reporter construct has been described in [74]. The ORF1-tRFP, Flag-ORF1p, Flag-eIF4AI, HA-Vpx_mac_, Myc-SAMHD1(human), HA-SAMHD1(human), Myc-SAMHD1(mouse), Myc-G3BP1 and Myc-TIA1 cDNA sequences were cloned into the pcDNA4 expression vector (Invitrogen). The Myc-SAMHD1 mutations H233A, D311A and H206A/D207A (named HDAA) were generated using PCR-based mutagenesis method. The wild type and mutated EGFP-SAMHD1 DNA sequences were cloned into pEGFP-C1 (ClonTech). Anti-Myc antibody (CST2276) was purchased from Cell Signaling, anti-HA.11 antibody (MMS-101P) from Covance, anti-SAMHD1 antibody (TA502085) from ORIGENE, rabbit anti-G3BP1 antibody (13057-2-AP), rabbit anti-TIA1 (12133-2-AP), rabbit anti eIF4G (15704-1-AP), and anti-actin antibody (60008-1-Ig) from Proteintech, mouse anti-G3BP1 antibody (clone 14E5-G9, 05–1938) from Millipore, goat anti-TIA1 antibody (SC-1751) from Santa Cruz, Alexa fluor 594-labeled goat anti-rabbit antibody (A11012), Alexa fluor 647-labeled goat anti-rabbit antibody (A21244), Alexa fluor 594-labled donkey anti-mouse antibody (A-21203) and Alexa fluor 594-labled donkey anti-goat antibody (A-11058) from Lifetech, Dylight 405-labled anti-rabbit antibody (072-08-15-06) from KPL. Anti-Flag M2 affinity gel (A2220) was purchased from Sigma, protein G agarose from Millipore. Calyculin A (9902) was purchased from CST.

### Production of ORF1p antibody

The cDNA of ORF1p was amplified by PCR and cloned into the expression vector pET28a (Novagene, Germany). Recombinant ORF1p was expressed after induction with IPTG and purified using Ni-NTA resin (QIAGEN) in accordance with the instructions from the manufacturer. The purified ORF1p was used to immunize rabbits. Rabbit serum was collected, purified via affinity chromatography (Abmart, Inc) and tested for their recognition of ORF1p in western blotting and immunofluorescence staining.

### Cell culture and transfection

The human embryonic kidney 293T (HEK-293T) cells, HEK-293 cells and HeLa cells were grown in the Dulbecco's modified Eagle's medium (DMEM) supplemented with 10% fetal bovine serum (Invitrogen). Cells were transfected with PEI (Sigma) or Lipofectamine (Invitrogen) according to the manufacturer’s instructions.

### Cell proliferation assay

HeLa cells were cultured in 96-well plates at a concentration of 1 × 10^3^ cells per well and transfected with plasmid DNA or siRNA oligos using Lipofectamine (Invitorgen). The cells were incubated for 48 h before examined with the CellTiter 96 AQueous One Solution Cell Proliferation Assay kit (G3582, Promega). Briefly, One-fifth of the volume of CellTiter 96 Aqueous One Solution was added into each well, cells were incubated for 4 h at 37°C. The absorbance of each well at 490 nm was measured using an ELISA reader (Multiskan EX, Thermo Scientific).

### LINE-1 retrotransposition assay

HEK-293 cells or HeLa cells were first transfected with CMV-L1-neo^RT^ reporter DNA with or without SAMHD1 DNA [44]. After forty-eight hours, cells were detached from the plates with trypsin digestion and seeded into 6-well plates at serial dilutions. G418 (0.75 mg/ml for HeLa; 0.6 mg/ml for HEK-293) was then added to select for resistant colonies [71]. Twelve days after selection when sizes of colonies were optimal for counting, cells were fixed with methanol at room temperature for 10 min and stained with 0.5% crystal violet (in 25% methanol) for 10 min as described in [79]. Number of cell colonies was determined for each transfection at a dilution that yielded fewer than 100 colonies. The LINE-1 EGFP reporter construct was utilized to examine the effect of arsenite treatment on LINE-1 retrotransposition as described [74]. Briefly, cells were transfected with LINE-1 EGFP reporter construct DNA. Six hours after transfection, cells were treated with arsenite (1 μM) for 48 hours. Five days after transfection, GFP-positive cells were scored by flow cytometry. As a control, cells were transfected with the pEGFP-C1 vector DNA that constitutively expresses EGFP.

### Quantification of reverse transcribed LINE-1 DNA by PCR

HeLa cells were transfected with CMV-L1-neo^RT^ reporter DNA with or without SAMHD1 DNA. Seventy-two hours after transfection, total cellular DNA was extracted with the QIAamp DNA Mini kit (QIAGEN). The same amounts of DNA template (250 ng) were subject to PCR with primers 5’-CAGTTCGGCTGGCGCGAGGCC-3’/ 5’-CAGTTCCGCCCATTCTCC G-3 that amplify reverse transcribed LINE-1 cDNA [46]. The first primer 5’-CAGTTCGGCTGGCGCGAGGCC-3’ crosses the exon/intron junction within the neomycin resistance gene such that only the spliced and reverse transcribed DNA can be amplified. Levels of β-globin DNA were determined using primers 5’-TATTGGTCTCCTTAAACCTGTCTTG-3’/ 5’-CTGACACAACTGTGTTCACTAGC-3’ [80], the data were used to normalize the amount of LINE-1 DNA. The DNA products were separated in 1% agarose gels and visualized with Ethidium Bromide staining.

### Quantification of LINE-1 RNA by RT-qPCR

HeLa cells were transected with CMV-L1-neo^RT^ reporter DNA, with or without SAMHD1 DNA. Thirty-six hours later, total RNA was extracted using RNeasy Mini Kit (Qiagen). Equal amount of total cellular RNA was treated with DNase to remove the potential contamination of plasmid DNA. The RNAs were dissolved in diethylpyrocarbonate (DEPC)-treated water and reverse transcribed with 200 U SuperScript III Reverse Transcriptase (Invitrogen) according to the manufacturer's instruction using the oligo dT primer. The following primers were used to amplify LINE-1 ORF1 RNA: 5’-AAAGGAAAGCCCATCAGACTAACA-3’/5’-GAATATTGGCCCCCACTCTCT-3’. We also utilized a second pair of primers to quantify RNA that is transcribed from the reporter construct CMV-L1-neo^RT^. These primers (5’-CTGAAGCGGGAAGGG ACTG-3’ and 5’CCTTGAGCCTGGCGAACAG -3’) span the Neo cassette intron of the transfected L1 construct such that only spliced LINE-1 RNA is amplified to avoid the contamination by CMV-L1-neo^RT^ DNA. The actin mRNA was amplified with primers 5’-TGACGTGGACATCCGCAAAG-3’ and 5’-CTGGAAGGTGGACAGC GAGG-3’. The results served as internal controls. Quantitative RT-PCR was conducted in triplicates and each reaction underwent 40 cycles of amplification using an Applied Biosystems 7300 real-time PCR system (Applied Biosystems). Dissociation curves were generated for each sample to verify the amplification of a single PCR product. The relative expression was analyzed using the SDS 1.4 software (Applied Biosystems) [81].

### siRNA knockdown

The siRNA oligos and control siRNA were purchased from GenePharma (Shanghai, China). siRNAs targeting SAMHD1 mRNA are siRNAsamhd1-1 (CCTCGTCCGAATCATTGAT) and siRNAsamhd1-2 (GCAGCTG ATTCGAGTATAT). G3PB1 siRNAs include siRNAg3bp1-1 (GGGCTTCTC TCTAACAACA) and siRNAg3bp1-2 (ggacaaatcagagcttaaa). siRNA targeting TIA1 include siRNAtia1-1 (ccgctccaaagagtacata) and siRNAtia1-2 (ggctaacagaacaactaat). Cells were seeded into 6-well plates one day before two sequential transfections with siRNA oligos (30 nM) using Lipofectamine RNAiMAX (Invitrogen). The knockdown efficiency of siRNA was examined by western blotting.

### Immunofluorescence microscopy

Cells were grown on glass coverslips before transfection with indicated plasmid DNA. Forty-eight hours after transfection, cells were fixed with 4% paraformaldehyde (in 1x phosphate-buffered saline) for 10 min at room temperature followed by a 10 min permeabilization with 0.5% TX-100 at room temperature. Cells were then incubated for 2 h at room temperature with indicated antibodies, anti-G3BP1 (1:1,000 dilution), anti-TIA1 (1:500 dilution) and anti-ORF1p (1:500 dilution). Alexa Fluor 647-labeled goat anti-rabbit antibody, Alexa Fluor 594-labled donkey anti-goat antibody, Alexa Fluor 594-labled donkey anti-mouse antibody and Alexa Fluor 594-labeled goat anti-rabbit antibody (1:1,000 dilutions) were used as secondary antibodies. Confocal images were acquired at room temperature using a Leica TCS SP5 (Leica Microsystems) mounted on an inverted microscope (DMI6000; Leica Microsystems) with an oil immersion 63x/NA1.4 objective lens (HCX PL APO CS; Leica Microsystems). All subsequent analysis and processing of images were performed using the LAS AF software (Leica Microsystems).

### Western blotting

Cells or viruses were lysed in RIPA buffer (0.1% SDS, 1% Triton X-100, 1% sodium deoxycholate, 150 mM NaCl, 10 mM Tris [pH 7.5], 1 mM EDTA). Equal amounts of cell or viral lysates were separated in SDS-12% PAGE. Proteins were transferred onto nitrocellulose membranes (Whatmann). The membranes were probed with indicated antibodies, followed by incubation with IRDyeTM secondary antibodies (1:20,000). Protein bands were visualized on a LiCor Odyssey instrument, and quantified using ImageJ automated digitizing program (NIH).

### Immunoprecipitation

Cells were lysed in RIPA buffer and clarified by centrifugation. One milligram of cell lysate was incubated with 50 μl of anti-Flag M2 affinity gel (A2220 Sigma) or protein G-agarose and anti-Myc antibody for 4 h on ice. Beads were washed at 4°C three times with a RIPA buffer. In order to investigate if the association between ORF1p and G3BP1 is RNA dependent, cell lysates were treated with 100 μg/mL of RNase A (Qiangen) at 37°C for 30 min before immunoprecipitation. The bound proteins were separated in SDS-12% PAGE and further analyzed by western blotting. To isolate RNA from immunoprecipitated materials, RNA was extracted with the QIAamp RNeasy Mini kit (QIAGEN), the poly(A) mRNA was further isolated using the PolyA Tract mRNA isolation system III kit (Promega). The LINE-1 transcript was measured using a RT-PCR protocol described previously [50,51]. Briefly, the first-strand of cDNA was synthesized with the ORF2 (-) primer (5’-CTG TGT CTT TTA ATT GCA GAA TTT AGT CC-3’). This cDNA was then amplified with forward primer (5’-AAG CTT GAA TTC GGG GGAGGA GCC AAG ATG GCC G-3’) and nORF2(-) primer(5’- TTA ATT AAT AGT CCA TTT ATA TTT AAA G -3’). The amounts of PCR products were further determined by quantitative PCR using the qL1F primer (5’-TTT CCA TCT GAG GCC TGC CCT AAA A-3’) and nORF2(-). The relative expression levels were analyzed using the SDS 1.4 software (Applied Biosystems). The PCR products also used in the second round of PCR that was performed with the nested forward (NF) primer (5’- GGA GCC AAG ATG GCC GAA TAG GAA CAG CT-3’) and nORF2(-) primer, the final PCR products were analyzed by gel electrophoresis. DNA products were extracted from the gel with the QIAquick Gel extraction kit (QIAGEN), and further sequenced to confirm their identity as the LINE-1 sequence.

### ImageStream flow cytometer (Amnis)

A high-throughput multispectral fluorometric technique was used to analyze granules in HeLa cells. Twenty-four hours post transfection, HeLa cells were fixed, permeabilized, and stained with anti-G3BP1 antibody and anti-rabbit fluor 405-conjugated secondary antibody (Jackson ImmunoResearch). Over 1x10^4^ cells were analyzed for each sample. Digital images were recorded on a multispectral imaging flow cytometer (ImageStream100; Amnis Corporation, Seattle, WA) as described in [82]. Briefly, fluorometric compensation was digitally calculated based on single-stain controls. The single cells (gated in R1) in focus (further gated in R2) were selected based on a digital plot of aspect ratio with area and of the gradient root mean square (RMS) of bright-field images, respectively. Then EGFP and G3BP1 positive cells were gated in R3. tRFP, EGFP and G3BP1 cells were finally gated in R4 for granule counting and signal overlapping analysis. Following data acquisition, images were analyzed using the manufacturer’s software (IDEAS4; Amnis Corporation). A fluorescent punta was defined as a granule if this puncta is over 1 μm in size and has fluorescent signals 5-fold above the adjacent background. These threshold parameters were applied to the default mask of the representative cells according to the manufacturer’s instructions to determine the granule count. The degree of co-localization of two fluorescent signals was calculated as the bright detail similarity score feature using the IDEAS4 program.

### Operetta High-Content Screen

To quantify number of granules in cells, cells were visualized and analyzed on an Operetta High-Content Screen system (PerkinElmer). HeLa cells were transfected with indicated siRNA. Forty-eight hours post transfection, cells were seeded in triplicate at 20,000 per well in a CellCarrier-96 plate (6005550, PerkinElmer). 24h after, cells were fixed and permeablilized as described above and stained with DAPI and mouse anti-G3BP1 antibody overnight. After washing with phosphate buffered saline, cells were incubated at room temperature for an hour in the dark with goat anti-mouse AlexFluor 555. Plates were then scanned and images were collected with an Opertta HTS imaging system (PerkinElmer) at 20 × magnifications with 11 fields of view. Images were then analyzed with harmony software to quantify the mean number of granules per cell.

## Supporting Information

S1 Fig SAMHD1 inhibits LINE-1 retrotransposition. (A) HeLa cells or (B) HEK-293 cells were transfected with siRNA targeting SAMHD1 prior to transfection with the CMV-L1-neo^RT^ DNA. SAMHD1 levels were examined in western blots and the knockdown efficiency is determined by quantifying the intensities of SAMHD1 bands. Numbers of neomycin (G418)-resistant colonies were counted in three independent transfection experiments. The colony number of the control cells is arbitrarily set as 1. Images of a representative colony assay are shown. (C) HA-Vpx_mac_ DNA was used to deplete SAMHD1. Cells were co-transfected with Myc-SAMHD1 DNA and CMV-L1-neo^RT^ DNA, with or without HA-Vpx_mac_ DNA. Relative numbers of neomycin-resistant colonies of three independent experiments are shown in the bar graph. (D, E) HeLa cells (D) or HEK-293 cells (E) were co-transfected with Myc-SAMHD1 and LINE-1 reporter CMV-L1-neo^RT^ DNA. Neomycin-resistant cell colonies were scored and the results of three independent experiments are presented in the bar graph. Number of colonies from cells that had been co-transfected with the empty DNA vector pcDNA4 and CMV-L1-neo^RT^ DNA is arbitrarily set as 1. (F) HeLa cells were co-transfected with CMV-L1-neo^RT^ and the wild type human Myc-SAMHD1 or mouse Myc-SAMHD1. Numbers of neomycin-resistant colonies from three independent experiments are summarized in the bar graph. (G) HeLa cells were co-transfected with the CMV-L1-neo^RT^ DNA and the wild type Myc-SAMHD1 DNA or its mutants H233A, D311A and H206D207/AA. The H233A, D311A and H206A/D207A mutations alter the catalytic sites of SAMHD1. Expression of wild type SAMHD1 and its mutants were examined in western blots. Numbers of neomycin-resistant colonies from three independent experiments are summarized in the bar graph with the colony number of control cells (transfected by pcDNA4) is arbitrarily set as 1. (H) Levels of wild type SAMHD1 and its mutants were examined in western blots at day 3 and day 7 after transfection. * indicates *p*<0.05, ns denotes “not significant”.
(TIF)

S2 Fig Cell proliferation assay. (A) HeLa cells were transfected with siRNA targeting SAMHD1 prior to transfection with pcDNA3.1. Numbers of neomycin-resistant colonies from three independent experiments are summarized in the bar graph. The colony number of control cells is arbitrarily set as 1. (B) HeLa cells were transfected with pcDNA3.1 (carrying neomycin resistant gene) together with SAMHD1, TIA1 or G3BP1 DNA. Number of neomycin-resistant colonies with control cells is arbitrarily set as 1. Results shown are the average of three independent experiments. (C) HeLa cells were transfected with siRNA targeting SAMHD1. 72h post transfection, cells were examined with the CellTiter 96 AQueous One Solution Cell Proliferation assay kit. Results shown are the average of three independent experiments. (D) HeLa cells were transfected with SAMHD1, TIA1 or G3BP1 DNA. 72h post transfection, cells were examined with the CellTiter 96 AQueous One Solution Cell Proliferation assay kit. Results shown are the average of three independent experiments. (E) HeLa cells were cultured with the indicated concentrations of arsenite (AS) for 48 hours, and then examined with the CellTiter 96 AQueous One Solution Cell Proliferation assay kit. Results shown are the average of three independent experiments.
(TIF)

S3 Fig SAMHD1 diminishes the production of LINE-1 cDNA. (A) HeLa cells were co-transfected with CMV-L1-neo^RT^ and wild type Myc-SAMHD1 DNA or the SAMHD1-H233A mutant. Levels of LINE-1 RNA were determined by real-time RT-PCR using primers that either amplified the ORF1 RNA sequence (black bar) or the neomycin gene cassette (gray bar). Level of LINE-1 RNA in the control cells is arbitrarily set as 1. Expression of ORF1p and Myc-SAMDH1 was monitored in western blotting. (B) The reverse transcribed LINE-1 cDNA was measured by semi-quantitative PCR as described in Materials and Methods. Primers are designed to specifically amplify LINE-1 DNA that was reverse transcribed from the spliced neomycin resistant gene RNA. Semi-quantified PCR was performed with serially diluted DNA samples. Intensities of DNA bands were quantified, the results are summarized in the bar graph. Levels of β-globin DNA were also measured by PCR as internal controls.
(TIF)

S4 Fig Knockdown of TIA1 or G3BP1 inhibits the formation of stress granules induced by NaAs and SAMHD1. (A, B) HeLa cells were cotransfected with siRNA oligos targeting G3BP1 (A) or TIA1 (B). Twenty-four hours post transfection, cells were treated with arsenite (500 μM) for 30 min. Cells were stained with mouse anti-G3BP1 antibody and rabbit anti-TIA1 antibody. Nuclei were stained with DAPI (blue). (C, D) HeLa cells were cotransfected with siRNA oligos targeting G3BP1 (C) or TIA1 (D) and plasmids encoding EGFP-SAMHD1. Twenty-four hours post transfection, cells were stained with rabbit anti-TIA1 antibody or anti-G3BP1 antibody. Bars represent 10 μm.
(TIF)

S5 Fig A speculative model to illustrate how SAMHD1 inhibits LINE-1 retrotransposition. LINE-1 ORF1p and ORF2p associate with LINE-1 RNA to form RNP complex within the cytoplasm [35,38,39]. This RNP complex enters the nucleus where LINE-1 RNA is reverse transcribed into DNA by a target-primed mechanism [34,40,41]. Alternatively, LINE-1 RNP can be localized in cytoplasmic stress granules and sequestered there [36,37,49]. SAMHD1 is able to promote stress granule formation likely by elevating eIF2α phosphorylation or disrupting the interaction of eIF4A with eIF4G. This activity of SAMHD1 enhances the sequestration of LINE-1 RNP within the stress granules and thereby reduces LINE-1 retrotransposition. SAMHD1 also acts by diminishing the level of LINE-1 ORF2p as reported in [27].
(TIF)
