# Supplementary figures and images for: SAMHD1 Inhibits LINE-1 Retrotransposition by Promoting Stress Granule Formation
Source: PLoS Genet. 2015 Jul 2;11(7):e1005367. doi: 10.1371/journal.pgen.1005367 (PMC4489885; doi:10.1371/journal.pgen.1005367)

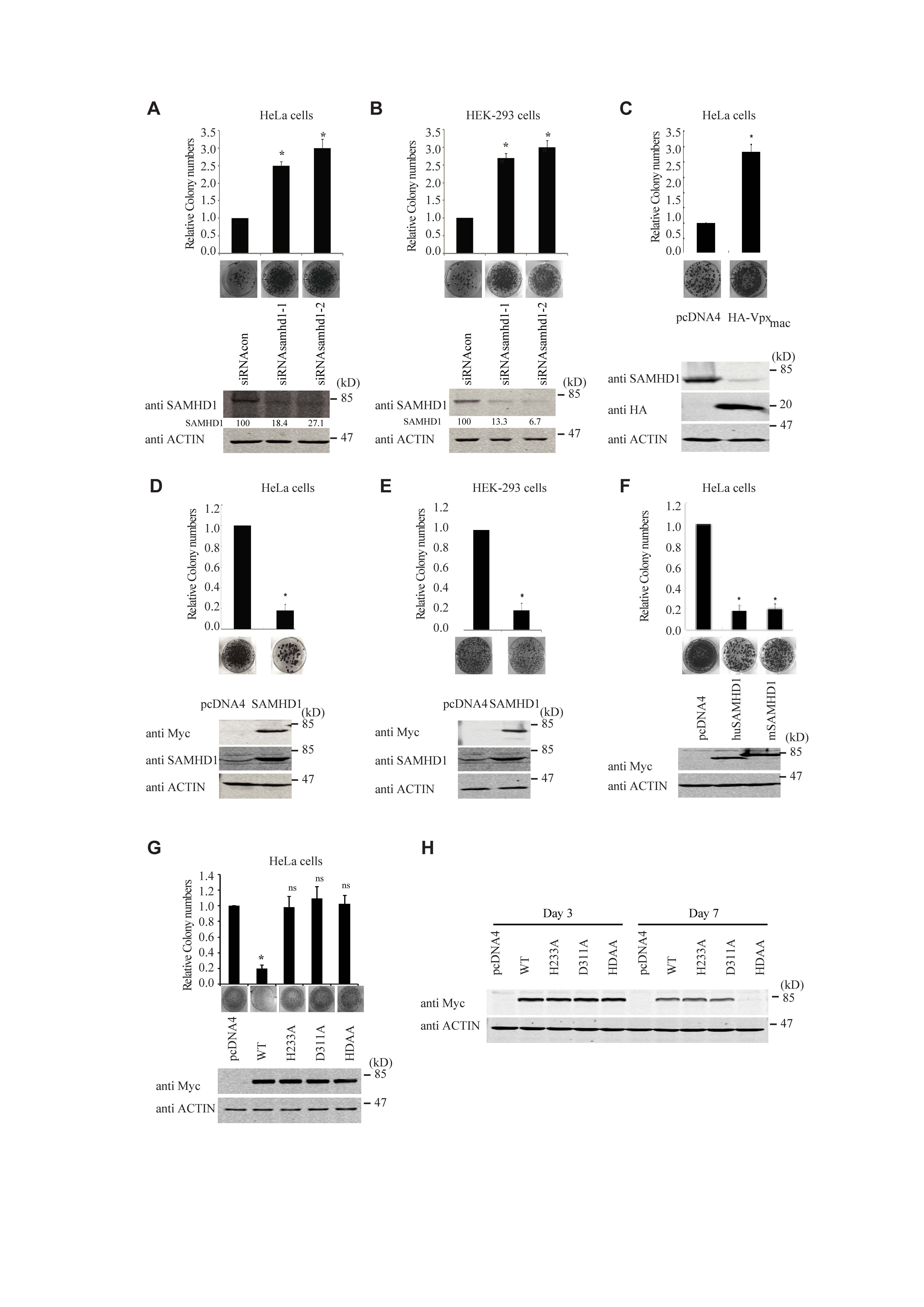

Supplement: S1 Fig — (A) HeLa cells or (B) HEK-293 cells were transfected with siRNA targeting SAMHD1 prior to transfection with the CMV-L1-neoRT DNA. SAMHD1 levels were examined in western blots and the knockdown efficiency is determined by quantifying the intensities of SAMHD1 bands. Numbers of neomycin (G418)-resistant colonies were counted in three independent transfection experiments. The colony number of the control cells is arbitrarily set as 1. Images of a representative colony assay are shown. (C) HA-Vpxmac DNA was used to deplete SAMHD1. Cells were co-transfected with Myc-SAMHD1 DNA and CMV-L1-neoRT DNA, with or without HA-Vpxmac DNA. Relative numbers of neomycin-resistant colonies of three independent experiments are shown in the bar graph. (D, E) HeLa cells (D) or HEK-293 cells (E) were co-transfected with Myc-SAMHD1 and LINE-1 reporter CMV-L1-neoRT DNA. Neomycin-resistant cell colonies were scored and the results of three independent experiments are presented in the bar graph. Number of colonies from cells that had been co-transfected with the empty DNA vector pcDNA4 and CMV-L1-neoRT DNA is arbitrarily set as 1. (F) HeLa cells were co-transfected with CMV-L1-neoRT and the wild type human Myc-SAMHD1 or mouse Myc-SAMHD1. Numbers of neomycin-resistant colonies from three independent experiments are summarized in the bar graph. (G) HeLa cells were co-transfected with the CMV-L1-neoRT DNA and the wild type Myc-SAMHD1 DNA or its mutants H233A, D311A and H206D207/AA. The H233A, D311A and H206A/D207A mutations alter the catalytic sites of SAMHD1. Expression of wild type SAMHD1 and its mutants were examined in western blots. Numbers of neomycin-resistant colonies from three independent experiments are summarized in the bar graph with the colony number of control cells (transfected by pcDNA4) is arbitrarily set as 1. (H) Levels of wild type SAMHD1 and its mutants were examined in western blots at day 3 and day 7 after transfection. * indicates p<0.05, ns denotes “not significant”. (T [file pgen.1005367.s001.tif]

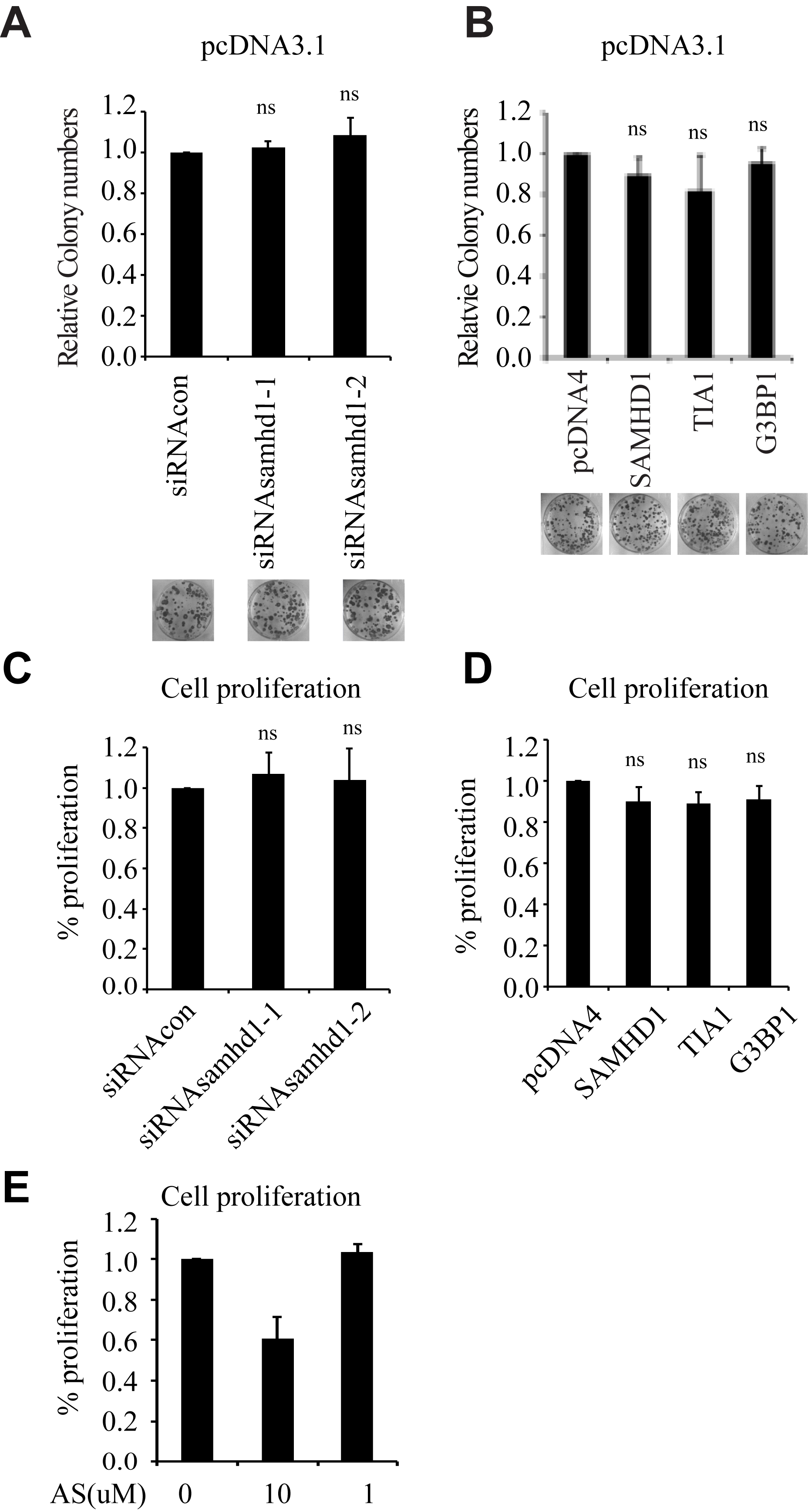

Supplement: S2 Fig — (A) HeLa cells were transfected with siRNA targeting SAMHD1 prior to transfection with pcDNA3.1. Numbers of neomycin-resistant colonies from three independent experiments are summarized in the bar graph. The colony number of control cells is arbitrarily set as 1. (B) HeLa cells were transfected with pcDNA3.1 (carrying neomycin resistant gene) together with SAMHD1, TIA1 or G3BP1 DNA. Number of neomycin-resistant colonies with control cells is arbitrarily set as 1. Results shown are the average of three independent experiments. (C) HeLa cells were transfected with siRNA targeting SAMHD1. 72h post transfection, cells were examined with the CellTiter 96 AQueous One Solution Cell Proliferation assay kit. Results shown are the average of three independent experiments. (D) HeLa cells were transfected with SAMHD1, TIA1 or G3BP1 DNA. 72h post transfection, cells were examined with the CellTiter 96 AQueous One Solution Cell Proliferation assay kit. Results shown are the average of three independent experiments. (E) HeLa cells were cultured with the indicated concentrations of arsenite (AS) for 48 hours, and then examined with the CellTiter 96 AQueous One Solution Cell Proliferation assay kit. Results shown are the average of three independent experiments. (TIF) [file pgen.1005367.s002.tif]

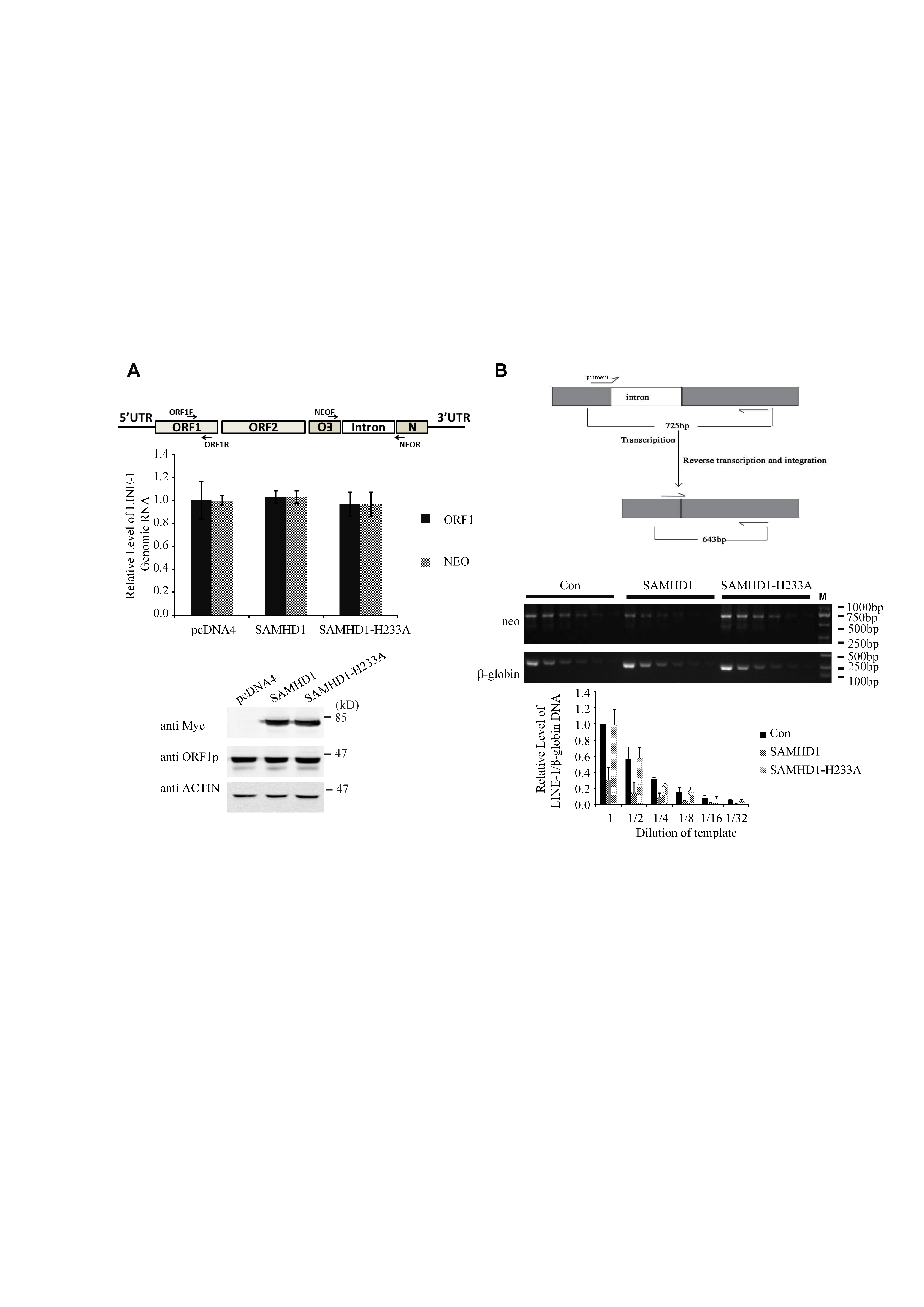

Supplement: S3 Fig — (A) HeLa cells were co-transfected with CMV-L1-neoRT and wild type Myc-SAMHD1 DNA or the SAMHD1-H233A mutant. Levels of LINE-1 RNA were determined by real-time RT-PCR using primers that either amplified the ORF1 RNA sequence (black bar) or the neomycin gene cassette (gray bar). Level of LINE-1 RNA in the control cells is arbitrarily set as 1. Expression of ORF1p and Myc-SAMDH1 was monitored in western blotting. (B) The reverse transcribed LINE-1 cDNA was measured by semi-quantitative PCR as described in Materials and Methods. Primers are designed to specifically amplify LINE-1 DNA that was reverse transcribed from the spliced neomycin resistant gene RNA. Semi-quantified PCR was performed with serially diluted DNA samples. Intensities of DNA bands were quantified, the results are summarized in the bar graph. Levels of β-globin DNA were also measured by PCR as internal controls. (TIF) [file pgen.1005367.s003.tif]

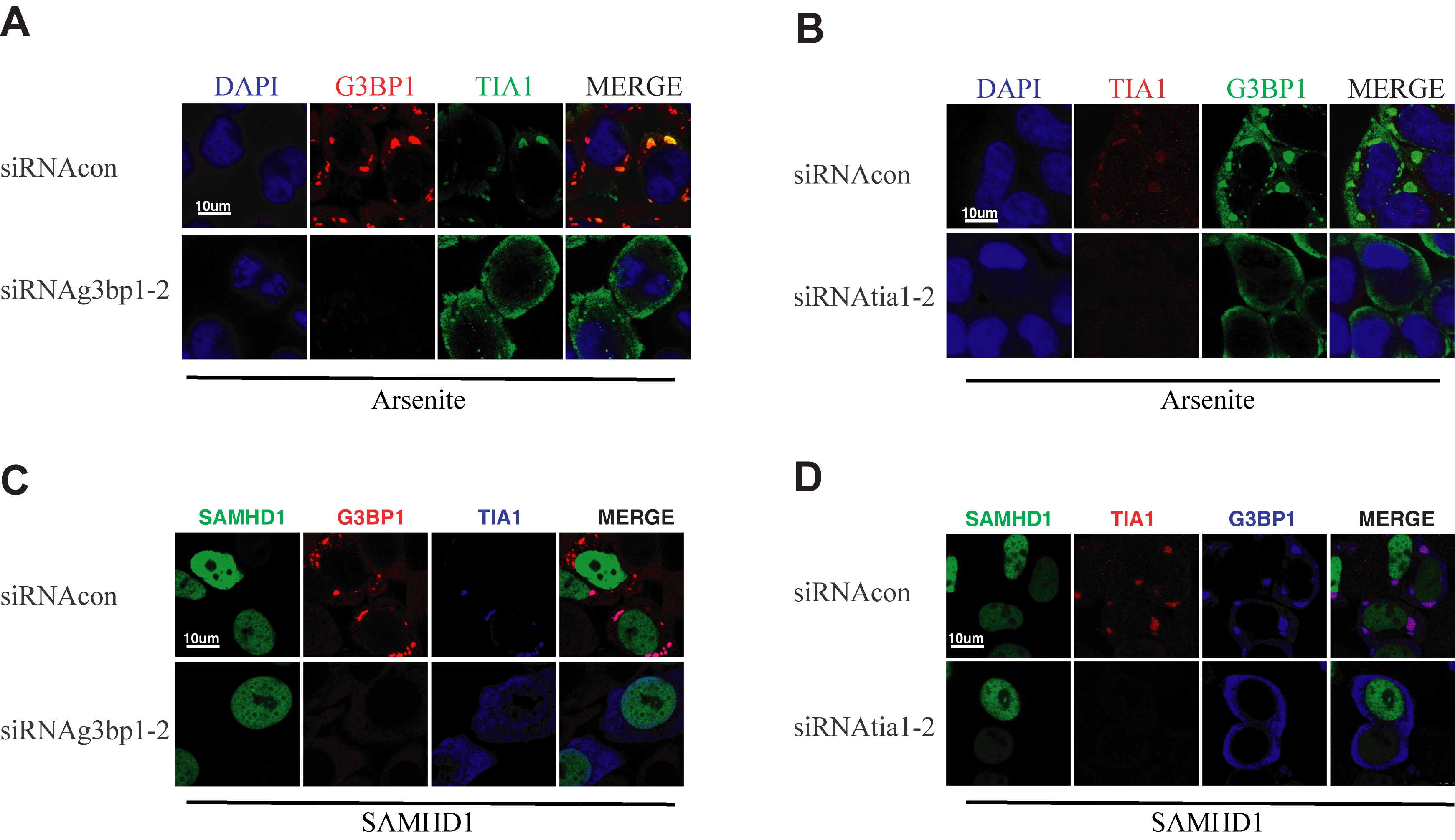

Supplement: S4 Fig — (A, B) HeLa cells were cotransfected with siRNA oligos targeting G3BP1 (A) or TIA1 (B). Twenty-four hours post transfection, cells were treated with arsenite (500 μM) for 30 min. Cells were stained with mouse anti-G3BP1 antibody and rabbit anti-TIA1 antibody. Nuclei were stained with DAPI (blue). (C, D) HeLa cells were cotransfected with siRNA oligos targeting G3BP1 (C) or TIA1 (D) and plasmids encoding EGFP-SAMHD1. Twenty-four hours post transfection, cells were stained with rabbit anti-TIA1 antibody or anti-G3BP1 antibody. Bars represent 10 μm. (TIF) [file pgen.1005367.s004.tif]

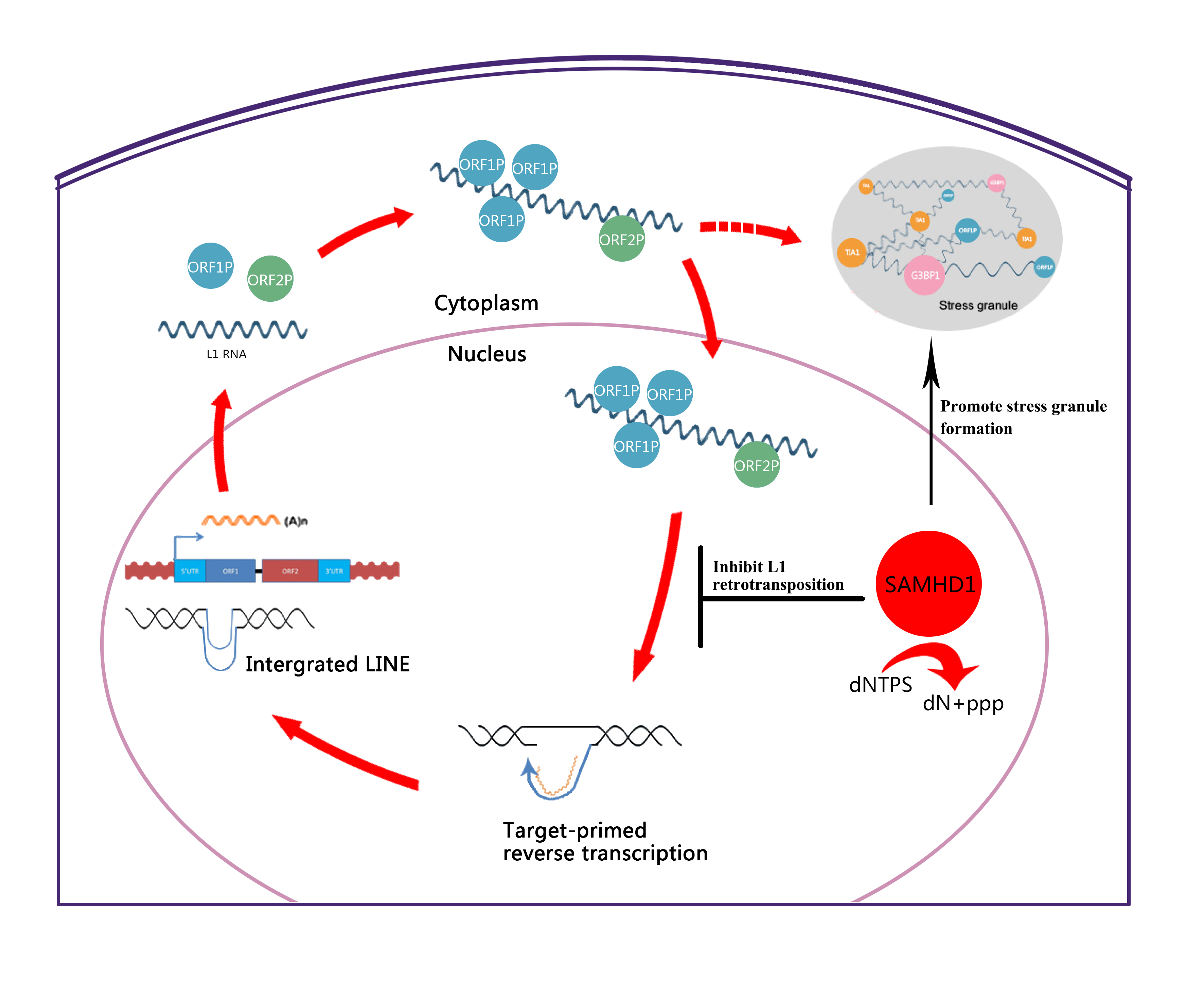

Supplement: S5 Fig — LINE-1 ORF1p and ORF2p associate with LINE-1 RNA to form RNP complex within the cytoplasm [35,38,39]. This RNP complex enters the nucleus where LINE-1 RNA is reverse transcribed into DNA by a target-primed mechanism [34,40,41]. Alternatively, LINE-1 RNP can be localized in cytoplasmic stress granules and sequestered there [36,37,49]. SAMHD1 is able to promote stress granule formation likely by elevating eIF2α phosphorylation or disrupting the interaction of eIF4A with eIF4G. This activity of SAMHD1 enhances the sequestration of LINE-1 RNP within the stress granules and thereby reduces LINE-1 retrotransposition. SAMHD1 also acts by diminishing the level of LINE-1 ORF2p as reported in [27]. (TIF) [file pgen.1005367.s005.tif]
